# All-optical generation of static electric field in a single metal-semiconductor nanoantenna

**DOI:** 10.1038/s41377-023-01262-8

**Published:** 2023-09-19

**Authors:** Yali Sun, Artem Larin, Alexey Mozharov, Eduard Ageev, Olesia Pashina, Filipp Komissarenko, Ivan Mukhin, Mihail Petrov, Sergey Makarov, Pavel Belov, Dmitry Zuev

**Affiliations:** 1 School of Physics and Engineering, ITMO University, Lomonosova 9, Saint Petersburg, 191002 Russia; 2 Center for Nanotechnologies, Alferov University, Khlopina 8/3, Saint Petersburg, 194021 Russia; 3 Higher School of Engineering Physics, Peter the Great Saint Petersburg Polytechnic University, Politekhnicheskaya 29, Saint Petersburg, 195251 Russia

**Keywords:** Nanophotonics and plasmonics, Nonlinear optics

## Abstract

Electric field is a powerful instrument in nanoscale engineering, providing wide functionalities for control in various optical and solid-state nanodevices. The development of a single optically resonant nanostructure operating with a charge-induced electrical field is challenging, but it could be extremely useful for novel nanophotonic horizons. Here, we show a resonant metal-semiconductor nanostructure with a static electric field created at the interface between its components by charge carriers generated via femtosecond laser irradiation. We study this field experimentally, probing it by second-harmonic generation signal, which, in our system, is time-dependent and has a non-quadratic signal/excitation power dependence. The developed numerical models reveal the influence of the optically induced static electric field on the second harmonic generation signal. We also show how metal work function and silicon surface defect density for different charge carrier concentrations affect the formation of this field. We estimate the value of optically-generated static electric field in this nanoantenna to achieve ≈10^8^V/m. These findings pave the way for the creation of nanoantenna-based optical memory, programmable logic and neuromorphic devices.

## Abstract (web summary)

This paper shows theoretical and experimental results of the electric field-induced second harmonic generation in a single metal-silicon nanostructure for an optical-induced control of the *χ*^(2)^ susceptibility.

## Graphical abstract

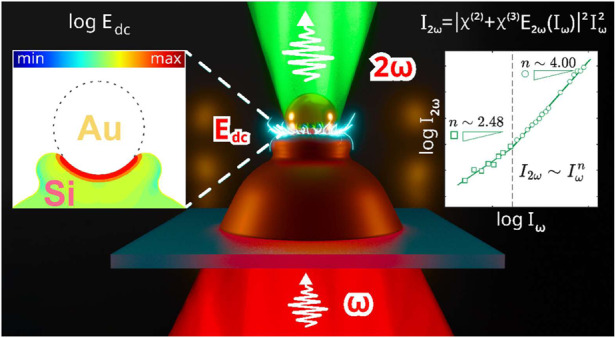

## Introduction

Electric field is a powerful tool for nanodevice engineering in solid-state nanoelectronics. It is applied to control logic operation by spin-orbit torque switching^1^ or ferromagnetism switching^2^, and to combine memory and logical operations in a single platform^3^. Moreover, electric field-controlled field-effect transistors are realized through quantum tunneling^4^, ferroelectricity^5^, self-heating plateau^6^, dynamic ionic gradients of metallic nanoparticle films^7^, and others.

While the technological progress of modern devices tends to the miniaturization and transition from operations with electrical signals to optical ones. This leads to the creation of integrated optical chips, which are a multicomponent photonic device on a single substrate^8^. The speed of information processing is mainly determined by the material response time. Thus, building elements (single nanoantennas) of potential optical chips can be modulated with pico and femtosecond time scale^9–11^, which makes such concept very promising in terms of computing speed. For this reason, the search for new approaches for fast optical signal processing determines the vector of modern research in photonics remaining for many years^12^.

In nanophotonics, local electrical field can be considered as a key to discover a wide range of nanoantenna features, originating both from solid-state material and optically resonant properties^13–16^. Such nanoantennas with double optical/electrical functionalities are vital for future nanophotonic processors and pave the way for nanoantenna-based neuromorphic devices. Therefore, the next challenging step in the development of nanophotonics is the controllable generation of electric field *inside* a single resonant nanoantenna.

In bulk semiconductors, an effective way for the creation of a static electrical field is using an interface (e.g., semiconductor-oxide or metal-semiconductor) for redistribution and separation of oppositely charged carriers. The impact of such a static electric field becomes obvious even in centrosymmetrical crystals such as silicon (the symmetry breaking occurs on the crystal surface), providing modulation of the effective value of second-order susceptibility *χ*^(2)^ through the interaction with the third-order one *χ*^(3)^^17,18^. And materials with a non-zero ${\chi }_{{\rm{eff}}}^{(2)}$ makes it possible to create optical frequency converters based on it, which performs the function of operating and controlling nonlinear optical signals (e.g., second harmonic one) for creation of high-speed reconfigurable nonlinear optical components of nanophotonic devices.

Currently, electric-field-induced second harmonic generation (EFISH) effect has been successfully used for ultrafast dynamical control over SHG signal behavior in various materials (2D materials, polymer, ZnO, etc.)^14,19^ and geometries^20,21^ by means of external bias voltage^20,22,23^. However, one point is that this SHG control by a bias voltage cannot take place on an ultrafast scale, because a current becomes unsustainable at high operating frequency under required high voltage^24,25^

Moreover, to reveal the full potential of advanced optical devices (e.g., optical chip), it is desirable to manipulate the SHG response on the pico-second and shorter time scale by the all-optical control^26,27^ through the optical-induced charge separation^28^. The achieved results make it likely that the huge potential of the EFISH effect can be discovered in a single resonant nanoobject. The design of such a resonant nanosystem is complicated, as it should include a source of electrons, interface for the charge accumulation forming a static electric field, and a centrosymmetric system providing a significant modulation of the effective value of *χ*^(2)^.

Here, we develop a nanoantenna with a built-in optically generated static electric field. For this, we design a resonant metal/high-refractive-index semiconductor nanostructure (MSN) with a pronounced metal-semiconductor interface, at which optically generated electrons create a static electric field. The nanoantenna geometry is tuned to switch off the resonant enhancement of the SHG signal originating from the silicon nanoparticle volume. Therefore in the proposed nanosystem, the SHG signal serves like a probe, being time-dependent and demonstrating a non-quadratic signal/excitation dependence when the static electric field is present. This unusual SHG behavior is explained by the influence of optically-induced static electric field, which is supported by theoretical calculations. To estimate the value of static electric field and influence of metal work function and surface density of silicon on the charge carrier concentration, we carry out theoretical studies. Our work demonstrates for the first time the creation and probing of optically-induced electric field in a single resonant nanoparticle. The presented results are highly promising for the creation of various nanoantenna-based systems for optical nanotransistors, memory and programmable logic devices.

## Results

### EFISH effect theory

In centro-symmetric materials (Si, Ge, WSe_2_, etc.), the second-order nonlinear susceptibility *χ*^(2)^ of the bulk crystal is zero in the dipole approximation due to the symmetry of the crystal structure. Thus, the SHG effect in these materials is forbidden. Therefore, to generate second harmonic signal in such a crystal, symmetry breaking is required. The crystal symmetry can be broken by introducing one of the following elements^29^: (1) crystal surface or interface, (2) crystal tension, or (3) static electric field. In the first^30,31^ and second^32^ cases, only a quadratic dependence of the SHG signal on the excitation intensity is observed. The dependence of the SHG signal on the excitation intensity becomes non-quadratic when the system is exposed to a static electric field *E*_*dc*_^17,18,28,33^.

The electrical-field-induced second harmonic generation (EFISH) effect allows modulation of the effective value of second-order nonlinear susceptibility *χ*^(2)^ through non-zero elements of the fourth-rank cubic nonlinear susceptibility tensor *χ*^(3)^ as indicated below:

1 $${I}_{{\rm{SHG}}}(2\omega )=| {\chi }^{(2)}+{\chi }^{(3)}{E}_{{\rm{dc}}}{| }^{2}{I}_{{\rm{exc}}}^{2}(\omega )\approx B\cdot {I}_{{\rm{exc}}}^{n}(\omega )$$

where *I*_exc_ and *I*_SHG_ are the intensity at the laser excitation frequency *ω* and the SHG signal intensity at 2*ω*, respectively.

The part of the expression in the modulus of Eq. (1) is usually called the effective second-order nonlinear susceptibility ${\chi }_{{\rm{eff}}}^{(2)}={\chi }^{(2)}+{\chi }^{(3)}{E}_{{\rm{dc}}}$. From this expression, we can conclude that in order to obtain a pronounced EFISH effect, it is necessary to choose materials with a low bulk *χ*^(2)^ and a relatively large *χ*^(3)^, like in centro-symmetric crystals. Due to this, a ${\chi }_{{\rm{eff}}}^{(2)}$ depending on the static electric field will make the major contribution to the formation of the SHG response. These conclusions made it possible to formulate a criterion for a material search and the nanosystem design based on the EFISH effect.

Currently, two methods exist for the creation of a static electric field in bulk samples: it can be induced by bias voltage^34^ and photo-generated free charge carriers^17,18,28,33^. In the latter case, for semiconductor nanoscale systems, the static electric field is created by charge separation due to transport difference for electrons and holes (for example, potential barrier, mobility, etc.). The static electric field is formed if the pump photon energy is high enough for the photo-generation of free charge carriers. This static electric field also depends on the concentration of the photo-generated carriers, and the field strength depends on the excitation intensity *E*_dc_ = *f*(*I*_exc_). Thus, the EFISH effect provides a non-quadratic behavior of *I*_SHG_ = *f*(*I*_exc_) dependence (*n* ≠ 2) in semiconductor nanostructures. To facilitate the approximation of experimental data, Eq. (1) can be simplified and expressed as an nth-order polynomial function and an amplitude *B*. Surface/interface carrier traps additionally affect the static electric field formation^18^. The trapped charges create a space charge on the surface, which has a long lifetime (tens of seconds) compared to the recombination time of electron-hole pairs. This effect is manifested as a time-dependent SHG signal intensity, with saturation time of tens of seconds, which depends on the excitation intensity^35^.

Thus, in Si structures, there are two conditions for demonstration of the EFISH effect: (1) generation of free charge carriers in Si due to absorption of photons with energy higher than the Si bandgap and (2) carrier transfer through the potential barrier at the semiconductor-insulator interface. These conditions can be fulfilled due to the multi-photon absorption of radiation with wavelength less than 800 nm^18,33^. Therefore, for Si nanostructures with a thin oxide layer (for instance, a nanosphere^30^) under fs-laser illumination at a wavelength higher than 800 nm (for instance, 1047 nm), EFISH effect cannot be realized. The non-quadratic behavior and the time dependence of the SHG signal intensity can be used to reveal the presence of a static electric field.

### Fabrication of Mie-resonant hybrid MSNs

**Fig. 1:**
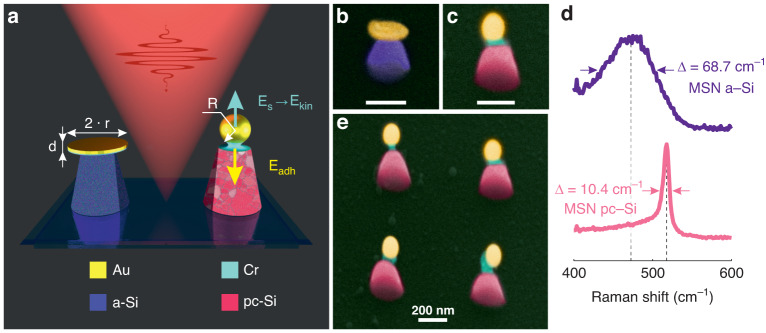
Laser-induced nanomechanics for the fabrication of MSNs. **a** Schematic of the processes defining the gold component modification. SEM images **b** initial hybrid nanoparticle, **c** MSN (scale bar of 200 nm). **d** Raman spectra of the hybrid nanoparticle before (violet curve) and after (pink curve) laser irradiation. Colors of the silicon component in the images indicate their crystalline state: violet means amorphous silicon, and pink crystalline silicon. **e** Array of the MSN

Design of a nanoantenna with built-in optical-induced electric field should include elements for efficient absorption of light inducing charge carriers and their transfer across an interface for charge separation. Therefore, in our experiments, to fabricate hybrid nanoantennas, we used metal-semiconductor nanoparticles (NPs) created with a combination of different lithography techniques (see the details in “Methods”). This approach allows the creation of ordered arrays of NPs consisting of gold nanodisks placed on the top of truncated silicon nanocones (Fig. 1a). It should be noted that the applied lithography methods are suitable for the creation of uniform arrays or hybrid NPs, but due to fabrication features, the dielectric components of the NPs are made of amorphous silicon. In turn, the presence of a nanocrystalline multigrain structure in Si NPs provides a well-developed net of interfaces, which enhances the SHG signal due to the dipole surface sources induced at interfaces between the grains^30,36^. To crystallize the silicon component, the hybrid NPs can be irradiated by fs-laser beams in pulsed mode. This method has proved to be efficient for selective crystallization of amorphous silicon NPs^37^.

To crystallize the Si component after the lithography stages, we used a Ti:Sapphire fs-laser (800 nm, 100 fs, Avesta Project) focused by an objective (×50, NA = 0.75) to a spot of 1.3 μm with a pulse repetition rate (RR) of 50 Hz. The array of hybrid NPs was irradiated in a point-by-point mode to carefully reconfigure each element. As expected, this fs-laser modification significantly changed the shape of the metal-semiconductor NPs: the Au nanodisk transformed into a nanosphere (NSph) and the NSph became partially detached from the nanocone, see the colored SEM images in Fig. 1b, c, e and the corresponding original SEM images in Fig. S1.

The process of metal NPs’ detachment (or jump) under irradiation by intensive laser pulses was observed and described for triangular gold nanostructures on a highly orientated pyrolytic graphite substrate^38^. Such a jump is related to a fast transformation of the surface energy (*E*_*S*_) into kinetic one (*E*_kin_) and an upward shift of the center of mass in NPs during their shape modification (Fig. 1a). The change in the surface energy Δ*E*_*S*_ of a flat gold disk as it turns into a detached droplet can be written as:

2 $${{\Delta }}{E}_{S}={\gamma }_{lv}((1-{\rm{cos}}\theta )\pi {r}^{2}+2\pi rd-4\pi {R}^{2})$$

where *γ*_*lv*_ is the liquid-vapor surface tension of Au (1.15 N/m)^39^; *θ* is the contact angle of liquid Au on glass (140°)^40^; *r* and *d* are the radius and thickness of the initial nanodisk structure (95 and 20 nm), respectively; and *R* is the radius of the detached droplet. If we neglect the evaporation process (i.e., *V* = *dπr*^2^ = $\frac{4}{3}\pi {R}^{3}$), the kinetic energy *E*_kin_ of a droplet with velocity *v* can be expressed as:

3 $${E}_{{\rm{kin}}}=\frac{\pi {r}^{2}d}{2}\rho {v}^{2}$$

where *ρ* is the density of Au (19.3 g/cm^3^ for solid and 17.2 g/cm^3^ for liquid^41^). Assuming that the conversion ratio from *E*_*S*_ to translational *E*_kin_ is around 20%^38^, the droplet velocity *v* can be written as:

4 $$v=\frac{1}{r}\sqrt{\frac{0.4{\gamma }_{lv}((1-{\rm{cos}}\theta ){r}^{2}+2rd-4{R}^{2})}{d\rho }}$$

From Eq. (4), we estimate that a gold droplet created after irradiating a hybrid NP with a fs-laser has a speed of 38 m/s. For this estimate, we used the following parameters of the hybrid NP: *γ*_*lv*_ = 1.15 N/m, cos *θ* = −0.766, *r*_disk_ = 95 nm, *d*_disk_ = 20 nm, *R*_droplet_ = 50 nm, and *ρ* = 17,200 Kg/m^3^.

In our case, despite the high speed the Au droplet develops, it is held in place (on the nanoantenna) by a chromium interlayer. This thin (2 nm) Cr interlayer is usually added to improve the adhesion between metal and semiconductor materials during lithography. Despite being thin, the Cr interlayer affects the heating process in the nanoantenna. Indeed, at a wavelength of 800 nm, the light penetration depth into Au is about 13 nm (the absorption coefficient *α*_Au_(800 nm) = 7.7089 ⋅ 10^5^ cm^−1^)^42^. Therefore, only the Au nanodisk is heated by laser radiation directly, while the underlaying Cr layer and the truncated Si nanocone are heated by conduction. Moreover, the three components of the hybrid nanoantenna are made of materials (Au, Cr and Si) with different melting temperatures (1064, 1907 and 1414 °C)^41^ and thermal conductivities (317, 93.7 and 148 W/m°K)^41^. For the jump process, an Au NP is heated to a temperature higher than its melting point, and it can even reach the boiling point (2856 °C)^38^, which allows the droplet to detach from the surface with a high speed. Such a fast detachment prevents the silicon component from overheating. Indeed, the SEM images show that the shape of the Si NP does not change under the selected irradiation conditions. In turn, Raman studies demonstrate a crystallization of Si component (see Fig. 1d). A typical broadband Raman peak of the amorphous Si, namely, a transversal optical (TO) phonon mode centered around 480 cm^−1^ is observed (Fig. 1d, violet line) for the hybrid nanoparticles before laser irradiation. After irradiation, a sharp peak at 520 cm^−1^, which corresponds to a TO phonon mode of poly-crystalline Si, is observed (Fig. 1d, pink line). Besides, a much lower full width at half maximum (Δ*ω*_*c*_ = 10.4 cm^−1^) is observed in the crystalline phase, compared to that of the non-irradiated hybrid nanoparticle (Δ*ω*_*a*_ = 68.7 cm^−1^). These facts suggest that the temperature reached under irradiation is higher than the Si crystallization threshold (around 650 °C)^43^ but remains lower than the Si melting point. In turn, the Cr layer partially melts and stretches between Au and Si, reducing the heat delivered from Au to Si and the bond between the Au and Si components in the hybrid nanosystem after fast cooling. Thus, the proposed fabrication approach is an efficient way for the creation of a hybrid nanosystem with a distinctive metal-semiconductor interface and components supporting Mie resonances.

In this metal-semiconductor nanoantenna, there are two main effects defining the second harmonic generation process related to the dimension choices. The first one is related to the metal-semiconductor interface contributing to the EFISH effect (for details, see Supporting Information, Fig. S8). The second one consists in the electromagnetic field concentration within the silicon cone volume due to the excitation of Mie resonances. Indeed, second harmonic generation signal was demonstrated to be increased by Mie resonances if their wavelength matches the excitation and/or the SHG signal wavelengths^30^. To minimize the SHG signal from the silicon volume, two criteria should be met: (1) non-resonant pumping of the system and (2) reduction of the Mie resonance impact on the silicon cone at the SHG wavelength. And to enhance the impact of the metal-semiconductor interface (i.e., of the static electric field generated by electrons) on the SHG process, the gold component of the nanoantenna should efficiently localize the field at the contact.

**Fig. 2:**
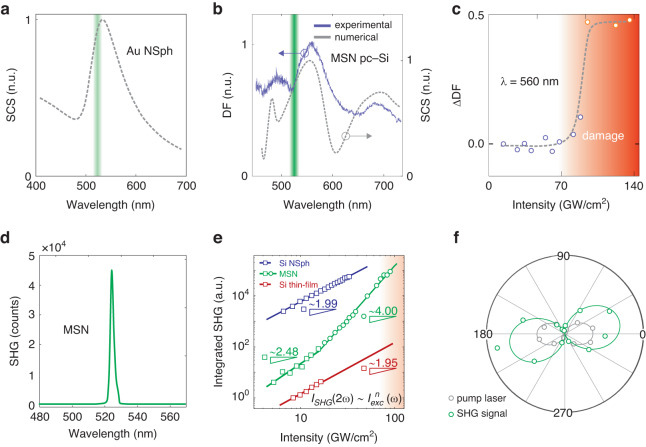
Design of a hybrid metal-semiconductor nanostructure for EFISH probing. **a** Numerical simulation of the scattering cross section of the Au nanosphere with a radius of 40 nm. **b** Experimental cross section (from dark-field spectrum) and the numerical scattering cross section of the MSN under oblique incidence of 68 degrees. Green lines in (**a**, **b**) indicate the wavelength of the SHG signal. **c** Monitoring the damage threshold of the MSN under fs-laser excitation through the dark-field spectra modification at 560 nm. **d** Typical SHG signal for the MSN at an excitation intensity close to the damage threshold. **e** Dependence of the SHG signal intensity *I*_SHG_ on the incident fs-laser intensity *I*_exc_ for the MSN, silicon nanosphere and a-Si:H thin film on a log-log scale. Solid lines: approximation of the experimental data by polynomial functions of the *n*th order (see the corresponding equation in inset of **e**). Triangles with numbers denote the slope of the approximating line in the log-log representation. **f** Polarization pattern of the laser beam and the MSN

Therefore, to utilize the resonant properties of the MSN components to study the SH signal in subsequent experiments, we slightly tune the geometry of the nanostructures (*D*_bot_ ≈ 270 nm, *h*_cone_ ≈ 110 nm) and perform fs-laser processing under the identical conditions. The scattering cross sections of the hybrid nanoparticle, obtained experimentally by confocal dark-field microscope (blue solid line) and numerically (gray dot line) in CST Microwave Studio, are demonstrated in Fig. 2b. The obtained spectra are in good agreement. Importantly, after the gold element transforms into a sphere, the resonant properties of the hybrid nanostructure are mostly determined by its silicon component^44^. In this case, resonant enhancement of the SHG from the Si component volume is avoided, which helps to highlight the EFISH effect from the hybrid nanoparticle. The resonance of the plasmonic component at ~523 nm (see Fig. 2a) localizes the electromagnetic field at the metal-semiconductor interface^44^, which enhances the SHG signal similar to previously considered hybrid systems^45^.

Thus, the designed hybrid nanoantenna uses Mie resonances to provide an efficient field localization at the Au/Si interface and avoid enhancement of the SHG signal from the silicon cone volume.

### SHG in hybrid MSNs

We studied the SHG effect for the MSN, using a resonant Si NP (125 nm in diameter) and a thin Si film (a-Si:H, thickness of 100 nm) as reference samples. We measured the SHG under the same excitation conditions as before, using the beam of a fs-laser operating at 1047 nm in the transmission geometry (see details in “Methods” and Supplementary Information, Fig. S2).

The SHG signal at 523 nm obtained from the MSN near its damage threshold is plotted in Fig. 2d. The SHG spectra of the MSN under increasing fs-laser excitation power are presented in Fig. S3a. We determined the damage thresholds of the MSNs by observing the changes in their scattering spectra while increasing the incident intensity step by step. The scattering spectra remained unchanged until the fs-laser intensity reached 95 GW/cm^2^, and at higher intensity values, they were dramatically modified (see the intensity difference at 560 nm in Fig. 2c and the original spectra in Fig. S3b). A similar approach was applied for the resonant Si NP. For the thin silicon film, the damage threshold was determined by visually observing the surface modifications in the area under irradiation. Thus, we found the damage thresholds for the MSN, Si film, and resonant Si NP to be 95, 15 and 35 GW/cm^2^, respectively. The damage threshold corresponds to thermal overheating of the structure^46^ due to heat accumulation and consequent melting of the sample, while before the threshold no modification in optical response is observed. This is because fs-laser heating saturates in a picosecond time-scale and does not undergo significant changes on a second time-scale. Thus, we can conclude that the contribution of temperature to the SHG time-dependence observed in the experiment is negligible. The *I*_SHG_ = *f*(*I*_ex_) dependence for the thin Si film has a standard quadratic dependence. For a more comprehensive comparison, we have repeated the same SHG experiments for a polycrystalline Si NSph fabricated by fs-laser ablation of a-Si:H film on a silica substrate at room conditions (for fabrication details, see article^30^). The printed Si NSph possesses a pronounced magnetic dipole resonance at the SHG wavelength (see Fig. S4a). As Fig. 2e shows, Si NSph has SHG with a quadratic dependence, as predicted.

On the contrary, the *I*_SHG_ = *f*(*I*_ex_) dependence for the MSN (see Fig. 2e) is non-quadratic. The fabrication method presented in this work enables reproducible fabrication of MSNs with an acceptable deviation from the average geometric parameters (see Fig. S5a–f). The repeatability of the fabrication and the non-linear SHG response are confirmed by SEM images and optical experiments. To prove this, five MSNs were chosen (Fig. S5b–f). The SHG signals were collected from each single MSN independently (see Fig. S5g). The SEM images and optical experiment show that the MSN shapes and the SHG signals are well-reproducible (as well as the non-quadratic behavior of the SHG). Also, in the case of our experiments we have checked the stability of the MSN morphology upon excitation of the SHG signal via the changes of a dark-field scattering signal. Indeed, the geometry and material properties of a nanostructure affect how it scatters light. Therefore, changes in the morphology of the MSN can be traced in the dark-field scattering spectra. The measurement cycles of the same MSN are demonstrated in Supplementary Materials (please see Supplementary Information, Fig. S13: the time stability of the SHG signal under the constant excitation intensity as well as repeatability of the SHG signal/excitation intensity dependence with time-delay are demonstrated). The represented data shows that the MSN morphology is preserved during the generation of the second harmonic at the excitation power stopping short of the 70 GW/cm^2^.

The SHG polarization pattern from the MSN is compared with the laser one in Fig. 2f. The MSN polarization pattern has two symmetrical lobes with linear polarization. These lobes are similar to those of the laser radiation at 1047 nm, but they are rotated by a small angle of 12.7° with respect to the incident beam polarization. The polarization patterns for different MSNs demonstrate a similar rotation (see Fig. S6). This effect is likely to originate from the multi-grain structure of the Si component of the MSN. The same polarization dependence of the SH signal was comprehensively studied for all the angles of crystal lattice rotation in single zinc sulfide nanowires^47^.

Consequently, our MSNs demonstrate a distinctive intense SHG signal, and its intensity has a non-quadratic dependence on the pump power. Such a behavior is typical for the EFISH effect^33^. Thus, the developed hybrid nanoantenna is an effective system for the detection and research of the EFISH effect.

### Probing the EFISH effect in MSNs

The capture of free charge carriers on traps with an inherent characteristic lifetime directly affects the optically generated static electric field. For this reason, the field value and, as a consequence, the SHG intensity are time-dependent, which can be used as the second way to detect the field existence (the first one is non-quadratic SHG behavior).

**Fig. 3:**
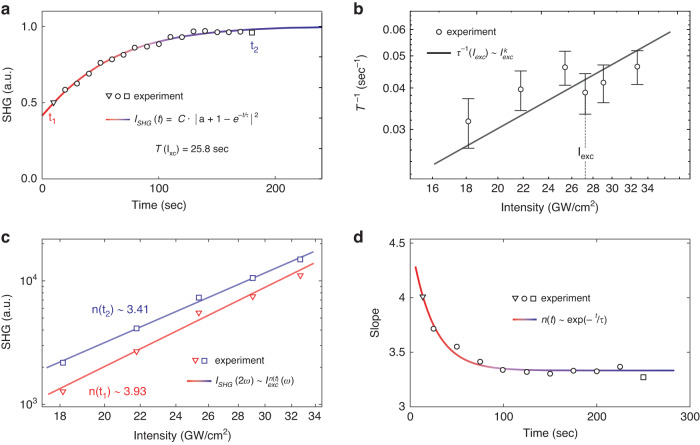
Time-dependent SHG signal in MSNs. **a** Time-dependent SHG signal under a laser excitation intensity of *I*_exc_ ≈ 26.7 GW/cm^2^. **b** Inverse characteristic electron trap lifetime (*τ*^−1^) (derived from the SHG signal) vs. excitation intensity (log-log scale). Solid line indicates linear fit in log-log scale. **c** SHG signal *I*_SHG_ of MSNs as a function of incident fs-laser power intensities *I*_ex_ in log-log scale at two different time moments. **d** Evolution of *n*(*t*), the slope of SHG signal vs. power in log-log scale, with time

In MSNs, the SHG response is time-dependent at a fixed pump intensity: the SHG signal increases for tens of seconds and then saturates (see Figs. 3a and S7 for different excitation intensities). A similar effect was observed and studied for a bulk intrinsic Si(001) sample with a defect layer (silicon oxide, thickness less than 5 nm) under fs-laser pumping in the wavelength range of 750–810 nm^17^. According to this work, such a time-dependence is explained by trapping and detrapping of free charge carriers at the defect interface rather than changes in the linear properties of the structure, thermal or recombination processes. The SHG signal dynamics in the MSN can be described by an adapted equation for the time dependence of the SHG process in bulk silicon^17^ as follows:

5 $${I}_{{\rm{SHG}}}(2\omega )=C\cdot {\left\vert a+1-{\rm{exp}}\left(-\frac{t}{\tau }\right)\right\vert }^{2}$$

where *a* is a time-independent dimensionless term that indirectly contains the initial density of trapped charges, *C* is the parameter taken from the saturation conditions for *I*_SHG_(*t* → *∞*) → 1, and *τ* denotes the electron trapping lifetime. This equation is the solution of the differential rate equation in semiconductors, and it describes the model of the time-dependent SHG signal^35^. We should note that this equation is derived supposing that the parameters *a* and *τ* depend on the pump intensity: *a* = *f*(*I*_exc_) and *τ* = *f*(*I*_exc_).

To further investigate how the SHG signal slope (in log-log scale) changes with excitation intensity, we use an *n*-order polynomial time-dependent function (see Eq. (1)). Figure 3c shows the dependence of the SHG intensity on the laser intensity in a log-log scale chosen for two time moments (*t*_1_ and *t*_2_, also marked in Fig. 3a), i.e., for an increasing SHG signal (*t*_1_ = 25 s) and a saturated one (*t*_2_ = 250 s). Similar slopes are plotted for the all time moments from Fig. 3a and shown in Fig. 3d, summarizing the time-dependence of the SHG slopes in log-log scale. This dependence exponentially decays from *n*(*t* = 0) = 4.5 to the saturated level *n*(*t* = *∞*) = 3.4. When the saturation level is reached, all the dynamic processes associated with charge transfer are stopped. This saturation level is maintained while the SHG excitation continues.

For comparison, we performed time-dependent SHG experiments for the fs-laser ablated polycrystalline Si NSph. We observed a stable behavior of the SHG signal over time (*I*_SHG_ ≠ *f*(*t*)) for different power intensities (see details in Supplementary Information, Fig. S4b). In our experiments, the photon energy is lower than the Si-insulator potential barrier. Thus, no charges can cross the Si-insulator interface, and there is no time modulation of the SHG signal. Therefore, for a Si NSph, SHG signal retains a quadratic dependence (see details in Supplementary Information, Fig. S4a).

Electron trapping lifetime depends on the excitation power. Figure 3b depicts an approximation of the experimental results (see Supplementary Information, Fig. S7) plotted in Fig. 3a by Eq. (5) for the inverse characteristic trap lifetime (*τ*^−1^) as a function of excitation intensity. Our results show that the inverse value of the trap lifetime increases with excitation intensity. The obtained values are consistent with the typical saturation time of time-dependent SHG, which depends on the excitation intensity, at the interface of a bulk semiconductor (tens of seconds)^17,48^. The slope of this dependence in log-log scale is defined by the absorption type in the considered system^17,48^. In our case, in silicon, at 1047 nm under the considered excitation conditions there is both one and two-photon absorption, as the absorption coefficients of these processes are comparable^49^. Therefore, the slope of the *τ*^−1^ = *f*(*I*_exc_) dependence in log-log scale is expected to be around 1.8. Our experimental data in Fig. 3b show the best convergence at ~1.2. This result can be explained by a Schottky barrier at the metal-semiconductor interface formed in the hybrid nanoantenna. Indeed, in contrast to pure silicon, the gold component in the hybrid nanoantenna also contributes to the generation of free charge carriers under such an excitation.

## Discussion

### Role of Schottky barrier in the formation of EFISH effect

If we excite the surface of a Si sample by a fs-laser operating in the NIR range (in our case, at 1047 nm/1.18 eV), the EFISH effect cannot be realized. Our MSN, however, has a lower energy of the metal-semiconductor potential barrier, which enables the EFISH effect. To theoretically describe the experimental non-quadratic dependence of SHG on the pump intensity, we apply the drift-diffusion model for the flow of thermally generated charge carriers, taking into account the Fermi-Dirac statistics^50^. The numerical drift-diffusion model considers the SHG signal at the initial time moment (*t* = 0, see Fig. 3d), when there is no interaction with carrier traps yet.

**Fig. 4:**
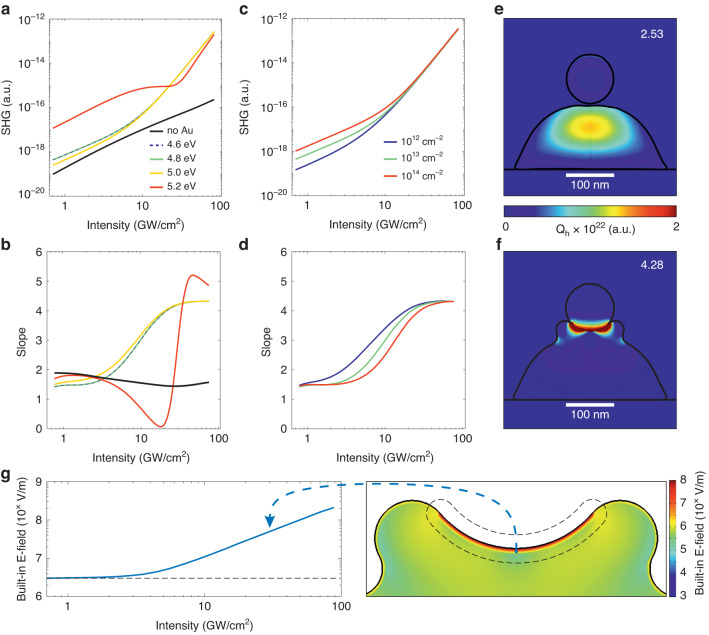
Numerical simulations of EFISH behavior. **a** Sum of EFISH signal as a function of the pulse intensity for the MSN on a log-log scale. Legend: metal work function of the metal part of the MSN. **b** Slope vs. excitation intensity for different metal work functions from (**a**). **c** Sum of EFISH signal vs. pulse intensity for different surface state densities. **d** Slope vs. excitation intensity for different surface state densities from (**c**). Absorbed power *Q*_*h*_ distribution for the MSN at a 1047 nm excitation by a plane wave from the bottom for MSN with (**e**) point metal-semiconductor contact and (**f**) a broad contact (buried gold nanosphere in the silicon truncated nanocone), respectively. **g** (left) The dependence of the static electric field on the excitation intensity at a point (marked by blue-dashed arrow) and (right) distributions of the static field from the numerical solution of the drift-diffusion model for the MSN in the semiconductor part at time of the peak excitation intensity (*t* = 0 fs)

The calculation results presented in Fig. 4a show the SHG signal vs. excitation power for the silicon nanocone with and without a gold nanosphere on a log-log scale. It is known that the work function for the adhesion Cr layer is 4.50 eV, and for the Au element, it is in the range of 5.10–5.47 eV^41^. The electron affinity of Si crystal surface is 4.05 eV. Therefore, the difference between the Au work function and Si affinity lies in the range of 1.05–1.42 eV, which is comparable with the excitation photon energy in the SHG experiments (1.18 eV). Thus, only the electrons that are able to absorb one or two photons can further cross the Schottky barrier and form the static electric field. For the hybrid MSN, the contact surface is composed of a thin Cr layer and an Au droplet. Thus, we should compare the experimental data with the calculations using the work function for the metallic component of the nanoantenna of 4.8–5.0 eV.

As can be seen from Fig. 4a, when the excitation pulse energy is below 7 GW/cm^2^, the slopes of log-log dependencies for all the work functions are around 2. In log-log scale, the SHG intensity vs. pump power calculated for the nanostructure with a metal work function of 5.2 eV slightly dominates over the dependencies plotted for metals with other work functions. A similar quadratic dependence is observed for the truncated Si nanocone without a metal component (see the black line). As the excitation intensity increases to 13–27 GW/cm^2^, the SHG signal dependence on the pump power for the truncated Si nanocone remains quadratic. In contrast, the MSN in this excitation range exhibits SHG with a nearly fourth-power dependence on the pump power for work functions in the range of 4.6–5.0 eV and a varied dependence for 5.2 eV (see Fig. 4b). In the experiment, for all the studied nanosystems, for low applied laser intensities, there is a small region with a “quadratic dependence” (*n* = 2), and at higher intensities, there is a “fourth-power dependence” (*n* = 4), as shown in Fig. 4b. These two power ranges are clearly observed in the experiments with SHG from the hybrid MSN (see Fig. 2e). There, two excitation regions in the log-log SHG signal/pump power dependence are observed, with the slopes of 2.48 and 4.0. For Si NSph, such a dependence is not observed, and the log-log slope is equal to 1.99 (see Fig. 2e), which is in good accordance with our theoretical predictions. Details of clear inspection on the variation of the SHG signal/excitation slope for the Si NSph within a small power range are demonstrated in Supporting Information, Fig. S4c.

On the other hand, within the high energy laser radiations, the silicon component is crystallized and an oxide layer is formed on its surface. The formed Si/SiO2 interface creates energy states in the band gap, which can be considered as spatially localized surface recombination centers (defects). These centers have a direct impact on the EFISH behavior, therefore in the case of the drift-diffusion calculations they are considered in the form of surface states with a discrete level lying in the middle of the band gap. Based on the numerical results, the two regimes were distinguished at low and high excitation intensity. A defective Si surface contains charge carriers traps with a density of 10^13^–10^14^ cm^−2^ at the energy lying in the middle of Si bandgap^51^ and metal work function of 5.0 eV. As can be seen from Fig. 4c, d, at low excitation intensities, the simulations show that the electric field is formed due to the localization of charge carriers in these states and does not significantly affect the SHG slope: the static electric field does not depend on excitation, and the behavior of the SHG is close to a quadratic dependence of signal vs. power (*n* ≈ 2). However, an increase in surface state concentration leads to a growth in the SHG intensity. However, when the pulse intensity exceeds 7–13 GW/cm^2^, a charge carrier density above 10^20^ cm^−3^ near the Au/Si interface is achieved at high excitation intensities. In this case a static electric field is created through the charge carrier separation at the Au/Si interface. The surface state density no longer affects the slope, and the static field created on the metal-semiconductor interface dominates. The EFISH signal demonstrates a fourth-power dependence instead of a quadratic one. The surface state density affects the SHG intensity at low excitation intensities and does not significantly change the SHG slope. However, an increase in surface state density leads to a shift in the power threshold existing between two specified regimes of the field creation as well as SHG intensity rise observed in the first excitation regime. This non-quadratic behavior originates from the processes near the Si/Au interface. The high absorbed power of the electromagnetic wave (see Fig. 4f) creates electron-hole pairs near the interface at a generation rate of *G*(*I* = 67 GW/cm^2^, *t* = 0) ≈ 10^35^ cm^−3^ s^−1^ (see details in “Methods“). This relatively high generation rate combined with slow recombination processes in the silicon part of the nanoantenna ensures the pumping level of electrons and holes *n*, *p* ≈ 10^20^ cm^−3^, which creates a strong separating electrical field near the metal-semiconductor interface. Thus, as follows from our numerical analysis, at a pulse intensity lower than 7 GW/cm^2^, the main contribution to the SHG signal is provided by the electric field generated by the defects at the surface of the silicon component of the nanoantenna. In this case, the dependence of the EFISH signal on the excitation power is quadratic. When the intensity becomes higher than 7 GW/cm^2^, the dependence changes to non-quadratic. In this case, a significant injection of the photogenerated carriers across the Si/Au interface forms a static electrical field, providing the dominant contribution to the EFISH effect. Such a field is inhomogeneous over the volume of the semiconductor and is mainly localized at the edges. The solution of the drift-diffusion model makes it possible to construct the distribution of the static field at different excitation powers and at different times during the action of the laser pulse. It is observed that at threshold power values between two slopes, a field is formed along the MS contact. If we start monitoring the field value at one point in space directly near the contact, then its value dependent on the pump power is shown in Fig. 4g, right panel. As can be seen, up to a certain threshold, the field strength does not depend on pumping power up to 10 GW/cm^2^. However, as the threshold is overcome, the field begins to grow polynomially with increasing excitation and reaches a value on the order of 10^8^ V/m (see Fig. 4g, left panel). The value is limited from above by the threshold of destruction of the nanostructure. More information on the electric field distribution is represented in Supporting Information (Fig. S12).

### The role of metal-semiconductor interface configuration

Further, we will consider the spatial distribution of the absorbed energy (*Q*_*h*_) in the hybrid nanostructure under plane-wave excitation from the substrate side at 1047 nm. For this purpose, different configurations of the metal-semiconductor interface should be studied (see Figs. 4e, f and S8). For each configuration, we carried out numerical simulations within the drift-diffuse model to estimate the magnitude of the SHG signal slope. Figure S8a presents the first case, when only the silicon part is considered. In this case, the maximum of the absorbed energy is localized closer to the geometrical center of the semiconductor structure. The dependence of the SHG signal on the excitation power is quadratic. If a gold sphere is added above the silicon structure with a small gap between them (see Figs. 4e and S8b), the distribution of the absorbed energy in the Si component remains virtually unchanged. The SHG in this case demonstrates a slight deviation from the quadratic dependence, with a log-log slope of *n* = 2.53.

The situation changes dramatically when the metal component comes into contact with the semiconductor one, as shown in Fig. S8c. In this case, the absorbed energy is localized directly at the metal-semiconductor interface, and the log-log SHG slope achieves 3.42. An even higher value of the SHG slope (*n* = 4.12) is observed when the nanosphere is slightly pressed into the truncated silicon nanocone (see Fig. S8d). This result is in good agreement with the experimental slope value for the MSN presented in Fig. 2e.

The observed differences between the slopes in these cases are related to an increasing *Q*_*h*_ from the hot spots of the plasmonic component at the metal-semiconductor interface. Indeed, the MSN is excited by a plane wave from the substrate side. A localized surface plasmon resonance is created in the gold nanosphere, leading to a localization of the electromagnetic field in the silicon nanocone close to the metal-dielectric interface. When the nanosphere is not embedded into the nanocone (see Fig. S8c), the hot spots are predominantly formed in the gap between the MSN components. For a sphere integrated in the semiconductor, these hot spots are created inside the semiconductor (see Fig. S8d, e). The most extreme case is a gold nanosphere completely surrounded by the semiconductor, forming a core-shell nanostructure (see Fig. S8f). In this case, the *Q*_*h*_ localization at the metal-semiconductor interface is maximal, and the SHG slope achieves a value of 4.26.

Thus, the comparison of the metal-semiconductor configurations (Fig. S8c–f) shows that during a gradual integration of the gold nanosphere into the Si nanocone, the SHG slope increases significantly up to 4.3 (see Fig. 4f). Thus, the excitation field and energy absorption are closely related to the semiconductor-metal interface.

In this paper, we have demonstrated a nonlinear metal-semiconductor gold-silicon nanoantenna with a built-in static electric field created by photon-generated charge carriers. Proposed design is based on standard lithography approaches and fs-laser treatment at the final fabrication step which allows investigated nanostructure to integrate in a real device. We reveal that the dependence of the SHG signal on the excitation power deviates from a quadratic one, with log-log slope changing from 2.48 to 4.00, which is caused by the interaction of the static electric field with the third-order susceptibility in silicon. This observation is consistent with our calculations of the effective second-order susceptibility based on the drift-diffusion model. The estimated value of the generated static electric field in the hybrid nanoantenna modifying the SHG signal is ≈10^8^ V/m. We demonstrate a combination of linear and nonlinear absorption in charge carrier generation, using for detection the time-dependent SHG signal and its interaction with charge traps on the metal-semiconductor interface. Our models show a direct dependence of the SHG log-log slope on the energy absorbed at the metal-semiconductor interface, varying from 2.0–4.3. The demonstrated SHG signal difference in the MSN can achieve the values of 2 orders of magnitude with the presence of a static electric field created by photogenerated charge carriers, which is achieved by the variation of the excitation intensity. Thus, an excitation regime affects the static electric field presence and modulates the SHG output with a high modulation depth, which in perspective paves the way for ultrafast logical operation in a single nanoantenna.

This work is the first step toward the use of optically-induced static electric field *inside* resonant nanophotonic systems like nanoantennas. Such a crossroads of optics (SHG, photo-induced charge carriers generation) and solid-state physics (electrical field generation, migration of charge carriers) will inevitably provide nontrivial metasurface designs with expanded functionalities. The answer to the question how to tune and control optically-induced electrical field in a single nanoantenna creates a wide range of opportunities in different areas of modern science. For instance, the dependence of the ${\chi }_{{\rm{eff}}}^{(2)}$ on the static electric field is an efficient tool for modulation of non-linear response in on-chip optical systems^12,26,52,53^. Indeed, in our case the static electric field is created optically, which makes proposed MSN design interesting not only for current technologies (e.g., logical elements utilizing a net of electrodes) but also for advanced full optically-controlled nanophotonic devices. In the latter case each MSN can be independently excited by femtosecond laser and photogenerated carriers form the static electric field on the metal-semiconductor interface. Thus the EFISH effect can be all-optically controlled by the femtosecond laser for each unit independently rather than by directly applying a voltage. So, the represented application of solid-state physical features in the resonant optical element design lay a ground stone for the creation of the all-optical transistor on-chip, as a way to escape from the time-response-limited optoelectronic technologies of the top-gate type.

## Materials and methods

### Sample preparation and characterization

#### Hybrid nanodimers fabrication

We used e-beam lithography, metal evaporation, lift-off procedure and gas-phase chemical etching of thin a-Si:H films, with a thickness ≈200 nm, deposited on a fused silica substrate. For more fabrication details, see article^54^. The created nanodimers are Au nanodisks placed on truncated Si nanocones.

#### Femtosecond laser modification

A commercial Ti:Sapphire fs-laser (Avesta project) combined with motorized linear translators with air suspension (Aerotech Inc., USA) was used to generate laser pulses centered at 800 nm (*τ*_pulse_ ≈ 100 fs, RR = 50 Hz, 281 GW/cm^2^). Laser pulses were intensively focused by an Olympus UPLFLN40X Objective with a numerical aperture (NA) of 0.75 and ×40 magnification. Each laser pulse was incident on the Si nanocones from top and thus modified the Au components rapidly. A focused laser beam with a Gaussian beam profile was moved along one of the rows of the NPs’ array, using Aerotech translators to move the sample with respect to the beam fixed in space. Due to various thicknesses and thermal extensions of the Cr thin film between the Au sphere and Si nanocone, hybrid MSNs have similar, but not exactly identical morphology. We also applied different laser intensities to study the critical conditions for the fabrication of ideal hybrid MSNs.

#### Sample characterization

We verified the morphology of each modified NP with a scanning electron microscope (SEM, Carl Zeiss, Neon 40) with normal and 45° oblique detection.

#### SHG measurements

SHG signal was measured by a confocal optical microscope equipped with separate and coaxial excitation and detection channels. Detection and excitation channels were focused by infinity-corrected objectives *M* = ×50, NA = 0.42 (Mitutoyo Plan Apo NIR HR) and *M* = ×10, NA = 0.26 (Mitutoyo Plan Apo NIR HR), respectively. A Yb^3+^ fs-laser (TeMa-150, Avesta Project) centered at 1047 nm was used to excite SHG response. A compact spectrometer (ASP-75, Avesta Project) and autocorrelator (AA-20DD, Avesta Project) were used for monitoring laser pulses. An attenuator pair comprised of a Glan prism (GL10-A, Thorlabs) and a half-wave plate (WPMH10M-532, Thorlabs) was used to vary the output power of the pumping laser, which was measured in front of the excitation channel by a power meter (Ophir, Nova II). A FELH0550 filter (Thorlabs) was applied to filtrate the emitted laser light within the excitation channel, and a FESH0850 filter (Thorlabs) was used in the detection channel to prevent laser radiation from reaching the detector. The measurements were carried out at room temperature (294 °K) in air. By rotating the Glan prism in the detection channel as the analyzer, the polarization pattern of the laser beam and NPs were characterized. We performed spectral analysis of the optical response of the system using a monochromator (Horiba Jobin-Yvon LabRam HR800 with a 150 lines/mm diffraction grating) and a water-cooled CCD detector (iDus DU420A-OE) in the optical range of 400–1000 nm.

#### Dark-field scattering spectroscopy

A confocal dark-field scheme was applied to study the scattering properties and demonstrate the unchanged morphology of the hybrid MSNs. The sample was illuminated by a non-polarized light (HL-2000-FHSA halogen lamp, Ocean Insight) at an angle of 68°, which was focused by an infinity-corrected objective *M* = ×10, NA = 0.28 (Mitutoyo Plan Apo). The detection channel was equipped with an infinity-corrected objective *M* = ×50, NA = 0.42 (Mitutoyo Plan Apo NIR HR). The monochromator setup was used for spectral analysis of the optical response with a 150 lines/mm diffraction grating.

#### Numerical simulation of the Maxwell’s equations

We used COMSOL and CST Suite Studio to calculate E-field distribution at the excitation wavelength (1047 nm), as well as the absorbed power distribution and scattering-cross section spectra of single nanoparticles illuminated by a monochromatic plane wave in the visible wavelength range to study their resonance properties. For the optical calculations, we used the spectral dependencies of the refractive index and absorption for silicon and gold^55,56^, and the refractive index of glass was chosen to be 1.5. In silicon, an increase in the absorption coefficient due to two-photon ionization of electron-hole pairs was taken into account, according to refs. ^57,58^. The laser pulse at the beam waist was simulated by a plane electromagnetic wave incident from the side of the glass substrate with a power in the range of 10^8^–10^11^ W/cm^2^, which corresponds to the peak power of the laser pulse.

### Numerical simulation of the drift-diffusion model

For the numerical model, we selected the geometry of the semiconductor based on the cross section of the silicon nanoparticle of the optical model. On the nanoparticle surface, surface recombination of charge carriers on energy traps was taken into account. To simplify the calculation, a discrete energy spectrum of traps was chosen, containing one energy level located in the middle of the band gap. The density of states was chosen to be 10^13^ cm^−2^, and the probability of electron and hole capture was 1.5 ⋅ 10^−9^ cm^3^/s, which corresponds to the data from literature for a non-passivated silicon surface^59^. The difference between the work function of electrons in Au (5.10–5.47 eV) and electron affinity in Si crystal surface (4.05 eV) lies in the range of 1.05–1.42 eV, which is comparable to the photon energy used in the SHG experiments (1.18 eV). The metal ball on top of the silicon particle was taken into account by placing a metal electrode, an ideal Schottky barrier, on top of the nanoparticle. The work function of chromium is about 4.5 eV, and for gold, it is in the range of 5.10–5.47 eV^60^. Since the thickness of the chromium layer is about 2 nm, and the film might be non-continuous, the effective work function of the material in contact with silicon may be in the range between the work functions of chromium and gold.

The numerical calculation was carried out within the framework of the drift-diffusion model for the flow of thermally induced charge carriers in COMSOL Multiphysics, taking into account the Fermi-Dirac statistics^50^. The generation rate of charge carriers photoinduced in silicon was calculated based on the results of the optical problem in the following form:

6 $$\begin{array}{l}{G}_{{\rm{Si}}}({W}_{{\rm{pulse}}},t)=\frac{{Q}_{l}({W}_{{\rm{pulse}}},t)}{\hslash \omega }+\frac{{Q}_{nl}({W}_{{\rm{pulse}}},t)}{2\hslash \omega }\\ \qquad\qquad\qquad=\frac{{\alpha }_{{\rm{Si}}}({\lambda }_{{\rm{exc}}})\cdot {I}_{{\rm{exc}}}}{\hslash \omega }+\frac{{\beta }_{{\rm{Si}}}({\lambda }_{{\rm{exc}}})\cdot {I}_{{\rm{exc}}}^{2}}{2\hslash \omega }\end{array}$$

where *λ*_exc_ = 1047 nm is the excitation wavelength, ${\alpha }_{{\rm{Si}}}({\lambda }_{{\rm{exc}}})=\frac{2\pi }{{\lambda }_{{\rm{exc}}}}{k}_{{\rm{Si}}}({\lambda }_{{\rm{exc}}})$ is the absorption coefficient of silicon, ${\beta }_{{\rm{Si}}}({\lambda }_{{\rm{exc}}})=\left[\frac{{\rm{cm}}}{{\rm{GW}}}\right]$ is the two-photon absorption coefficient. The results of the drift-diffusion model are in good agreement with the experimental results if the value of the two-photon absorption is 18 $\frac{{\rm{cm}}}{{\rm{GW}}}$, which is greater than in the case of crystalline silicon^49^. This value can be explained by the formation on the surface of defects or silicon-metal alloy during lithography and femtosecond laser modification. *W*_pulse_ is the energy fluence of one ultrashort pulse, *Q*_*l*_ and *Q*_*nl*_ are the distribution of the absorbed power in silicon under one- and two-photon absorption, respectively, *ℏω* is the photon energy of the exciting light pulse (in J), and *τ*_pulse_ is the ultrashort pulse duration parameter, which is ~1.76 times less than the FWHM of the laser pulse and was chosen to be 85 fs for the further work. The pulse shape in time domain was described as follows:

7 $${I}_{{\rm{exc}}}(t)=\frac{{W}_{{\rm{pulse}}}}{2{\tau }_{{\rm{pulse}}}}{sec}{h}^{2}\left(\frac{t}{{\tau }_{{\rm{pulse}}}}\right)$$

The SHG intensity was estimated gradually: (1) the distribution of the absorbed power *Q*(*W*_pulse_, *t*) at one and two photon absorption was calculated, (2) the problem of drift-diffusion model in the nanostructure was solved to determine the distribution of the static electric field, (3) for each point in space, the parameter was calculated, which related the excitation intensity at a given point in space and the magnitude of the static field, as Eq. (1), and then it was integrated over the volume of the silicon part and considered as a SHG signal from the nanostructure. Such a simplified approach was used to obtain an estimated dependence of the SHG intensity on the excitation intensity at the single wavelength. This approach does not take into account the effect of the coherent field, as was considered in ref. ^61^. We performed a numerical calculation considering the effect of a coherent field, where the nonlinear polarizability was described for silicon (for details, see Supplementary Information, Fig. S14). The calculations take into account the relationship between the frequency-dependent fields both at the fundamental and second harmonic wavelengths. The trend of the SHG signal/excitation intensity dependence is preserved: in calculations by the two methods (with and without coherent field effect), the SHG slope changes from 2 to 4. Therefore, to study the nature of non-quadratic SHG at the single wavelength our simplified approach is relevant for the current MSN system. The characteristic value for semiconductor materials equal to 10^−19^ m^2^/V^2^ was used as the value of ${\chi }_{Si}^{(3)}$^21^. For the semiconductor, we performed the calculation in the time range from −1000 to 300 fs, where the zero value corresponds to the maximum intensity of the laser pulse. For a fixed value of the pulse power at each moment of time, the pulse power *I*_exc_(*t*) was calculated.

### Supplementary information

Supporting information
