# Supplementary material for: All-optical generation of static electric field in a single metal-semiconductor nanoantenna
Source: Light Sci Appl. 2023 Sep 19;12:237. doi: 10.1038/s41377-023-01262-8 (PMC10507031; doi:10.1038/s41377-023-01262-8)
Supplement: Supplementary file 1 — Supporting information [file 41377_2023_1262_MOESM1_ESM.pdf]

Supplementary information: All-optical generation of static electric field in a single metal-semiconductor nanoantenna

Yali Sun,[†,§] Artem Larin,[†,§] Alexey M. Mozharov ,[‡,¶] Eduard Ageev,[†] Olesia Pashina,[†] Filipp Komissarenko,[†] Ivan Mukhin,[‡,¶] Mihail Petrov,[†] Sergey Makarov,[†] Pavel Belov,[†] and Dmitry Zuev[*,†]

†*School of Physics and Engineering, ITMO University, St. Petersburg 197101, Russia*

‡*Center for Nanotechnologies, Alferov University, Khlopina 8/3, Saint Petersburg, 194021 Russia*

¶*Higher School of Engineering Physics, Peter the Great Saint Petersburg Polytechnic University, Politekhnicheskaya 29, Saint Petersburg, 195251 Russia*

§*These authors contributed equally to this work*

E-mail: d.zuev@metalab.ifmo.ru

Experimental setup description

The transmission scheme of our nonlinear experimental setup is shown in Fig. S2. To study the photon up-conversion process, a commercial Yb-doped solid-state ultra-fast laser centered at 1047 nm is applied as the excitation source. The laser pulse duration is 150 fs with 80 MHz repetition rate. The intensity of the laser beam is controlled by an attenuator comprised of two Glan prisms (Glan1, Glan2) and a super achromatic half-wave plate (Thorlabs, 600-2700 nm). The operation principle of the attenuator is the following: the polarization of

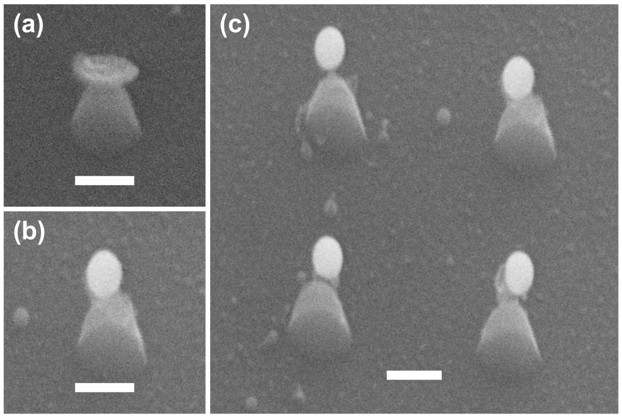

Figure S1: (a) SEM image of the initial hybrid nanoparticle. (b) SEM image of the MSN (scale bar is 200 nm). (c) SEM image of an MSN array.

the laser beam is selected by the first Glan prism (Glan1), then after passing the half-wave plate, the polarization of the laser beam rotates by a certain angle, and thus, it can not be fully transmitted through the second linear polarizer (Glan2), resulting in the attenuation of the beam intensity. Then, a power meter is utilized to measure the laser intensity excited to the system. To perform a pure excitation at 1047 nm and filter the green light, the laser beam passes through a long-pass filter (FELH1000, Thorlabs) and then is focused on the sample from the substrate side by a Mitutoyo Apochromatic NIR Objective with a $\times 10$ magnification (MY10X-823, Thorlabs). The generated SHG signal is collected by a Mitutoyo Apochromatic NIR Objective with a 50X magnification (MY50X-825, Thorlabs), and the excitation beam is filtered by a short-pass filter (FESH1000). It should be noted that a longer cut-off wavelength of the short-pass filter is more preferable, because silicon is able to generate white light in the visible range in addition to generation of second-harmonic waves. The scattering spectra of MSNs are excited by non-polarized light at an angle of $68°$, and focused by an infinity-corrected objective $M = \times 10$, $NA = 0.28$ (Mitutoyo Plan Apo) to demonstrate the unchanged morphology.

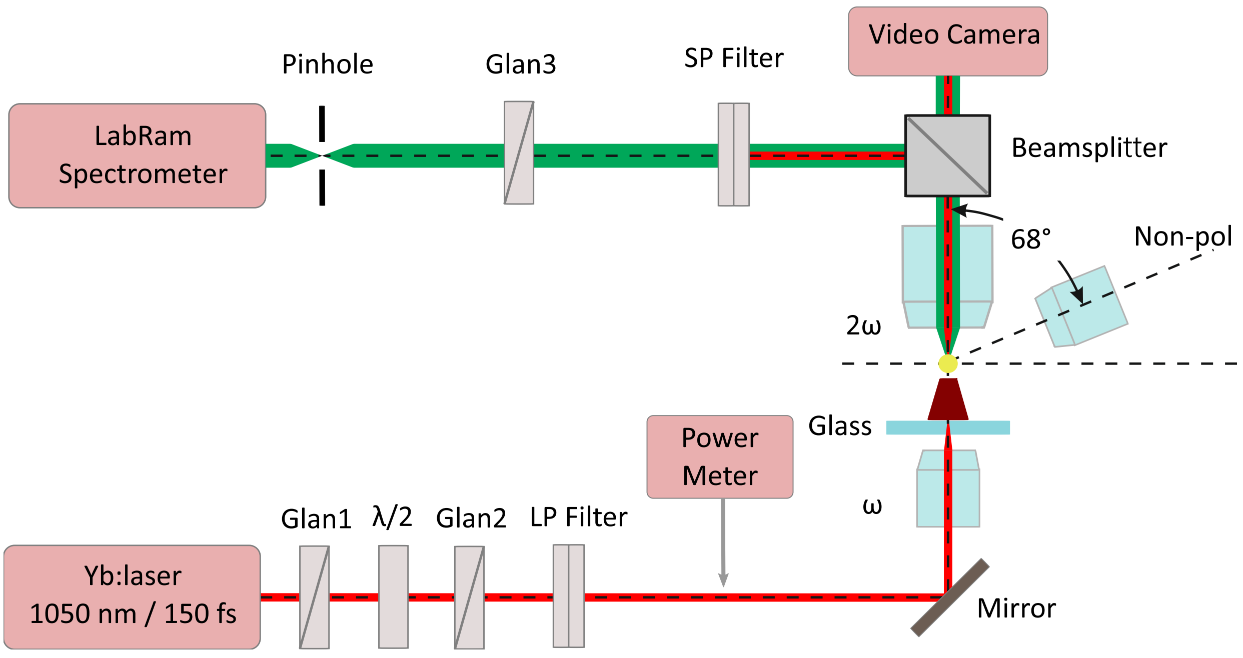

Figure S2: SHG and DF confocal microscope scheme

Damage threshold determination

Fig. S3 demonstrates the search process for the damage threshold of the MSNs. Fig. S3a shows the continuously increasing SHG signal from the MSN. Fig. S3b depicts the scattering spectra of the MSN in the initial state at 12 GW/cm^2, approaching the damage threshold at 77 GW/cm^2, and inflicted damage after 95 GW/cm^2. The scattering spectra first changes at 77 GW/cm^2: the intensity is reduced, but the resonance is preserved. After the excitation power exceeds 95 GW/cm^2, the pronounced resonance at 560 nm vanishes due to the destruction of the silicon nanocone. Fig.2c shows the intensity changing at the resonance (560 nm) for three stages. Blue dots indicate the unchanged scattering spectra and silicon nanocone morphology when the laser excitation intensity lies in the range of $12 - 77$ GW/cm^2. Red dots indicate the scattering intensity after the silicon nanocone is damaged.

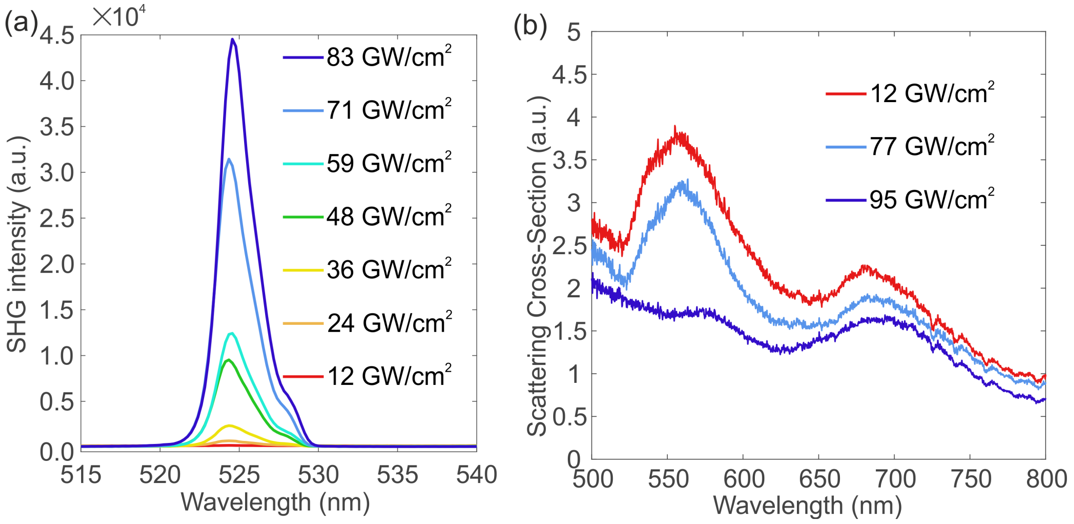

Figure S3: (a) SHG spectra of MSN with increasing fs-laser excitation power; (b) Scattering DF spectra of the MSN at several laser powers from (a).

Second-harmonic generation of the silicon nanosphere

Fig. S4 demonstrates the optical response of a silicon nanosphere. The particles were created by laser ablation of the a-Si:H thin film (100 nm) as a donor substrate to acceptor silica substrate at room conditions. Femto-second laser radiation (1047 nm, 150 fs, 1 kHz, 10 mW) was focused onto thin film by objective lens (NA = 0.26), and as a result, an array of separate nanospheres appeared on the silica substrate. To compare non-linear signal of the MSN with that of a purely dielectric structure, we selected a silicon nanosphere with a magnetic dipole resonance at SHG wavelength. From numerical simulation, we know that the diameter of the silicon sphere should be equal to 125 nm (Fig.S4a). To identify the silicon nanosphere size in experiment, dark-field spectroscopy was used (Fig.S4a). For such a silicon nanosphere, a quadratic dependence of SHG intensity on the laser intensity was shown in main text. It is in agreement with the report of Makarov et al.[1]. Fig. S4b shows time-independent SHG signal under different laser excitation intensities below the damage threshold. This typical silicon nanostructure is a good example for comparison with the hybrid MSN, highlighting the importance of metallic components.

The dependence of SHG on excitation intensity for a spherical sphere does not show a

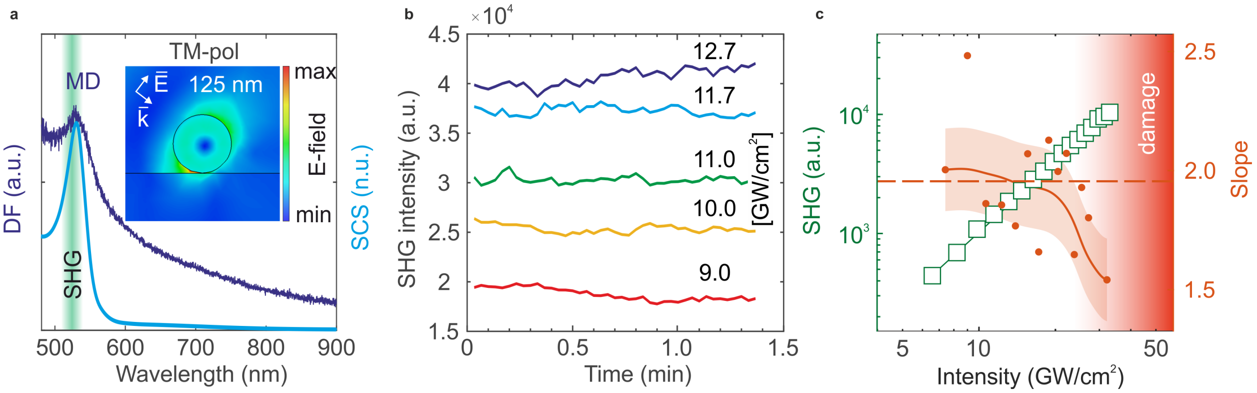

Figure S4: (a) Dark-field and scattering cross-section spectra for the silicon nanosphere on silica substrate, obtained experimentally and numerically, respectively. Inset: E-field distribution in Si nanosphere under plane-wave excitation at resonance wavelength (523 nm). (b) Time-dependent SHG signal of the silicon NP for excitation at 1047 nm for different values of average excitation power. (c) Experimental dependence of the SHG intensity and slope on the excitation intensity for a silicon nanosphere. Dashed horizontal line indicates SHG slope from linear approximation of the experimental dots (green squares) in the log-log scale. The solid red line is the result of smoothing the experimental data (red points) by the 'sgolay' method.

clear change in the SHG slope and can be linearly approximated on a log-log scale with a slope of ∼2 (Fig. S4c, horizontal orange dashed line). However, if we make a linear approximation between neighboring points, this will allow us to plot the dependence of the slope on the excitation power (Fig. S4c, red points). After a thorough result check, one can see that the slope decreases very slowly with increasing power. Perhaps, a more pronounced decrease of the slope could be observed if it were possible to obtain the SHG signal above the damage threshold.

Reproducibility of nanostructures

The fabrication method presented in this work makes it possible to create reproducible nanostructures with an acceptable deviation from the average geometric parameters (see Fig.S5a-f). The latter can be confirmed by an optical experiment determining the dependence of the SHG intensity on excitation intensity. To prove this, five nanostructures were chosen (Fig.S5b-f). They were obtained by the same method and studied the same way (see Fig.S5g).

The SHG signal was collected from each single nanostructure separately. To obtain statistics, for each pump power, we calculated the average value (marked with blue squares) and the standard deviation (range indicated by horizontal dashes) of the SHG intensity for five nanostructures. The optical experiment showed a good reproducibility of the SHG signal and the SHG slope.

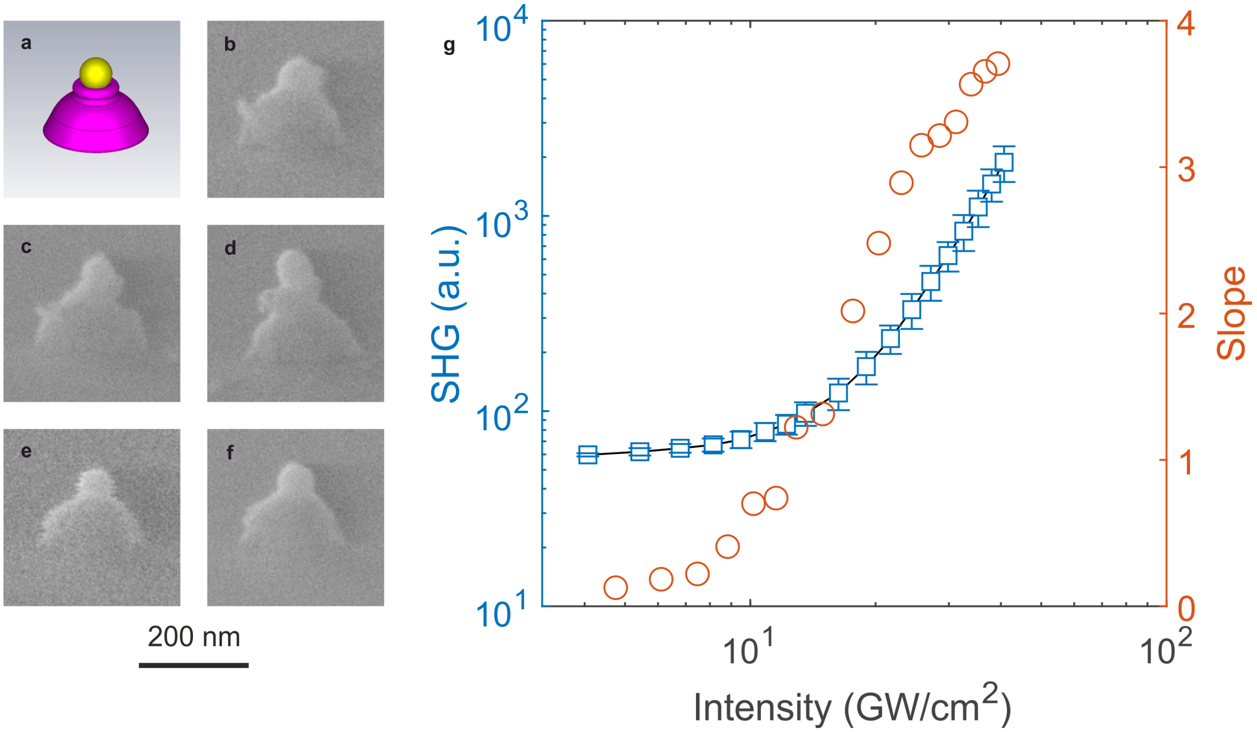

Figure S5: Fabrication yield and optical signal reproducibility. (a) Schematic of a typical MSN geometry, where the yellow sphere indicates the gold part and the pink object the silicon part. (b-f) SEM images of the MSN taken at 45° with respect to the normal of the glass substrate for several structures. (g) Dependence of the SHG intensity (blue squares) and of the slope (orange circles) on the excitation intensity.

Polarization-resolved second-harmonic generation

Fig. S6 demonstrates the excitation polarization pattern of the laser beam and SHG emission polarization pattern for MSN. The excitation laser beam is linearly polarized, exhibiting two symmetrical lobes (see Fig. S6a). SHG polarization patterns of MSN (Fig. S6b-c) demonstrate symmetrical shape similar to that of the excitation laser, but with different rotation

angles with respect to the laser polarization, which are marked on the top of each graph. The initial silicon nanocones fabricated by lithography are comprised of amorphous silicon, which is confirmed by Raman measurements. Further, these silicon nanocones are crystallized during their reshaping by fs-laser irradiation, and thus, they demonstrate an intense nonlinear signal. However, the polarization of the multi-grains interface is not fully controlled and shows different polarization lobes.

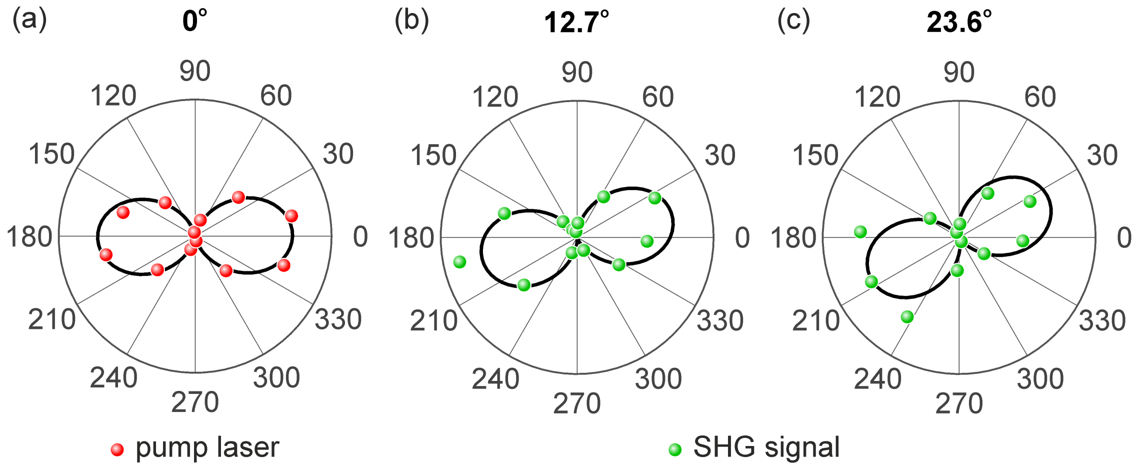

Figure S6: Measured polarization of excitation beam (a) and SHG emission from different MSNs (b, c). Solid lines indicate fitting with $SHG(\theta) = A \cdot sin^2(\theta + \phi)$; the value of ϕ is indicated in bold italics above each plot.

Time-dependent second-harmonic generation

SHG response of MSNs is time-dependent for each pump intensity: its signal rises for tens of seconds and then saturates. Fig. S7 shows the time-dependent SHG signal for different excitation intensities, which exponentially grows for tens of seconds.

The role of metal-semiconductor interface configuration

Fig. S8 shows the absorbed power distribution and SHG slope for nanostructures under plane wave excitation from the substrate side at 1047 nm. Dimension of the MSN–gold-

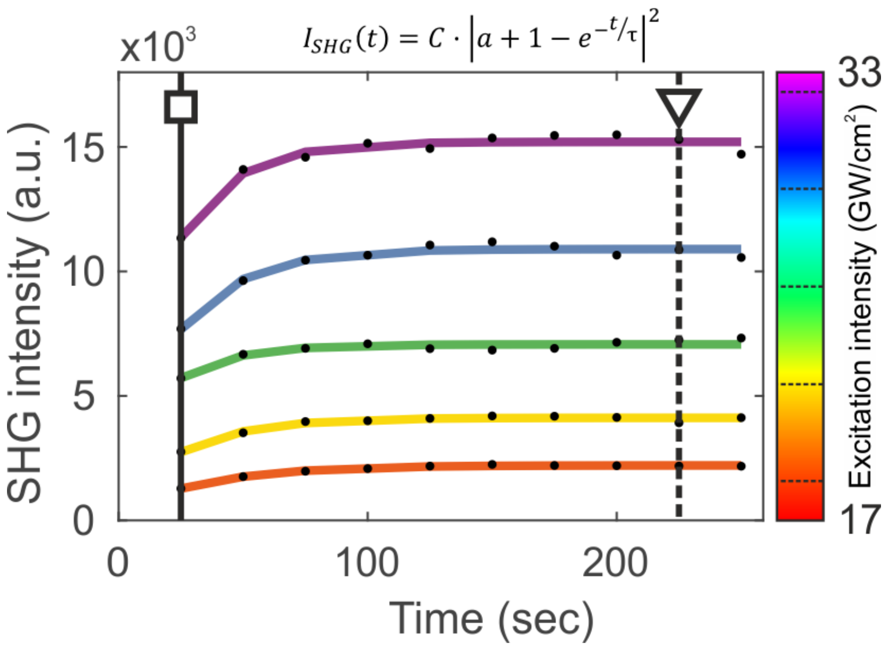

Figure S7: Time-dependent SHG signal $I_{SHG}(t)$ for MSN for different values of excitation intensity. The colors of the curves indicate the excitation intensity according to the color bar on the right.

silicon contact has an impact on the EFISH effect. As it was identified from our numerical simulation, the main criterion for increasing the EFISH contribution (i.e., the magnitude of the SHG slope) is the localization of the field directly at the metal-semiconductor contact (see Fig. S8. On the other hand, with a pure semiconductor component with a resonance or a tiny field confinement on the contact, the EFISH contribution as well as the SHG signal/excitation slope decreases (see Fig. S8 a-b).

In a single nanoantenna, the EFISH effect can be improved if resonances are used to localize the excitation field in the metal-semiconductor contact region. For this, the core-shell Au@Si nanoparticle and MSN are very well suited, because localized plasmon resonanse of the Au NSph helps to fullfile the criteria where the latter can be fabricated with resonances for the visible and NIR ranges.

The resonances of the structure are intentionally tuned so that the pumping laser wavelength does not match the intrinsic resonances of the semiconductor nanostructure. Thus, we avoid the case of field localization predominantly in the volume of the semiconductor part.

Fig. S9b (bottom) shows an example of the SHG slope change for the semiconductor out of and in resonance. In both cases, the field is significantly localized at the metal-semiconductor boundary (Fig. S9a, inset). In the resonant case, the localization of the field at the boundary is much higher, leading to the SHG signal increased by an order of magnitude in the considered power range (Fig. S9b, top). However, radiation is absorbed in the entire volume of the semiconductor particle. Presumably, this fact leads to a decrease in the SHG slope (Fig. S9b, bottom).

Extended results of the drift-diffusion model

Fig. S10 shows the SHG signal value vs. time for a metal work function of 5 eV. For a low excitation intensity ($I < 7\ GW/cm^2$), an almost symmetric SHG shape is observed, similar to that of the excitation laser pulse (see Fig.S10). With the pulse intensity increasing to

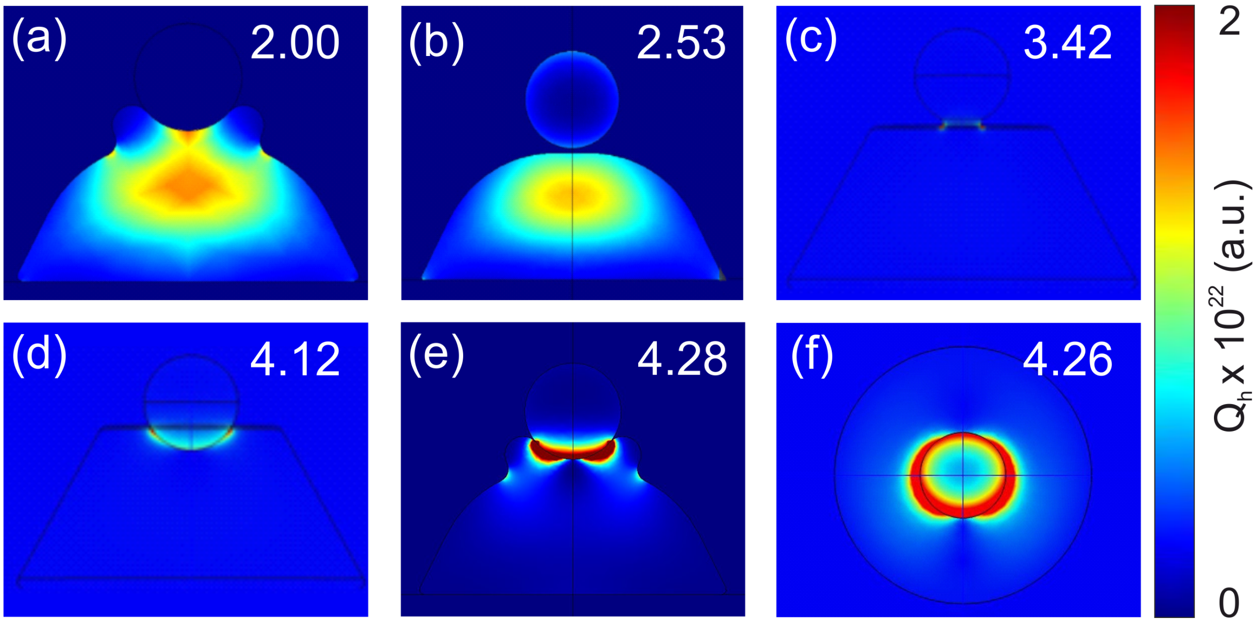

Figure S8: Absorbed power Q_h distribution for nanostructures under plane wave excitation from the bottom at 1047 nm. Excitation intensity is the same for all cases, as well as the color map. Numbers at corner indicate calculated SHG slope. The following cases are considered: no contact between the gold and silicon components (a), point contact (b), flat contact (c), embedded gold sphere (d), a geometry close to the experimentally observed one, and (e) metal core/dielectric shell system (f).

$I > 7\ GW/cm^2$, the SHG spectrum becomes asymmetric. This indicates the contribution of the separating photo-carriers to the SHG signal, as this process is delayed in time.

Fig.S11 shows band diagrams of the MSN (Fig.S11c) and carrier density (Fig.S11b) near the metal-semiconductor interface for various positions during interaction with an excitation pulse (90 GW/cm^2). For example, the work function of a metal is 4.8 eV. As can be seen, before the arrival of the exciting pulse (-1000 fs), there is a barrier for holes, which is determined by the relative positions of the work function of the metal and silicon with respect to the vacuum level. Obviously, the optical electromagnetic field increase leads to the electron-hole pair generations. Meanwhile, at early time (-300 fs), the photocarrier concentration is not enough to change the band diagram, including the height and width

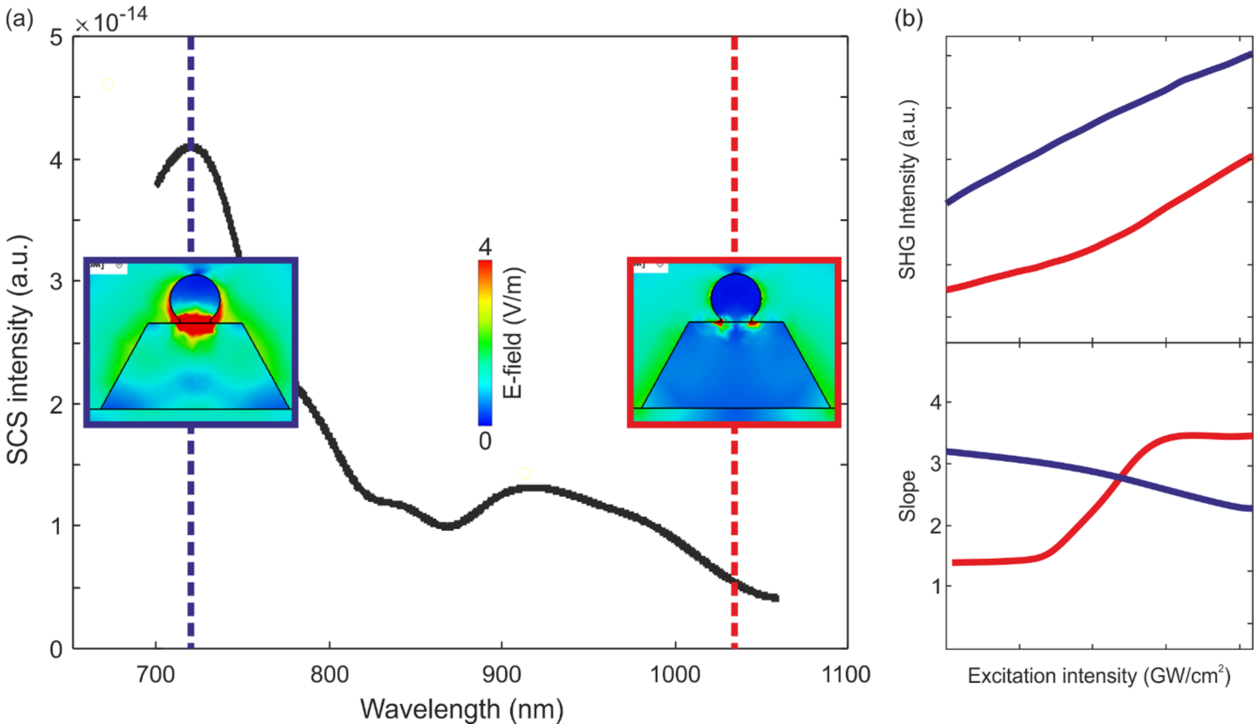

Figure S9: Role of the resonance in the SHG slope formation. (a) Numerically calculated scattering cross-section of the MSN (see nanostructure type at inset); color dashed lines indicate two cases, i.e. excitation wavelength out of (red) and in (blue) resonance with the intrinsic resonances of the silicon structure. Inset: Full E-field distribution for MSN in (top) and out of (bottom) resonance. (b) Numerical drift-diffusion model simulation for MSN nanostructure: (top) SHG intensity dependence on the excitation intensity in the log-log scale, (bottom) SHG slope dependance on the excitation intensity.

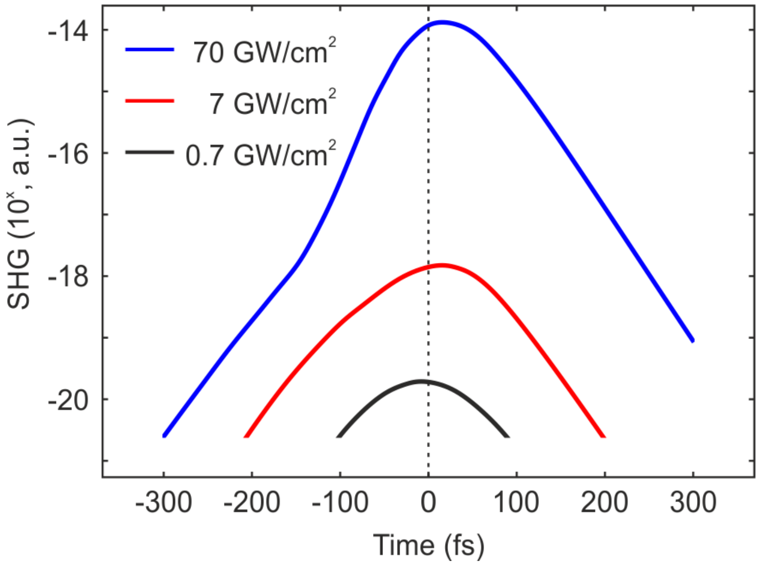

Figure S10: Magnitude of the SHG signal vs. time for the 5 eV metal work function.

of the barrier at the interface. It should be noted that for this time, an increase in the concentration gradient of charge carriers, both electrons and holes, near the interface is observed, which is associated with their active transition into the metal. At a first view, the hole transfer into the metal may not be obvious, since there is a barrier for them, but its height is too low to stop the holes. If we turn to the corresponding Fig.S11d for the current densities, we see that near the barrier, the diffusion current is several orders of magnitude greater than the drift current, which reduces the barrier impact on the total electron and hole currents. Charge carriers efficiently move into the metal by diffusion and recombine there. In turn, due to the higher mobility of electrons, the electron diffusion current is higher than the hole current, which leads to charging of the metal with an uncompensated negative charge. As a result, there is a rise in the right side of the band diagram and a decrease in the barrier for holes, clearly seen for -200 fs. By this moment, flat bands are formed, i.e., there is no electric field at the metal-semiconductor interface. This results in a decrease in the EFISH signal, as can be seen in Fig.S11a. Since the diffusion current dominates over the drift one, the flat bands do not affect the transfer of electrons and holes into the metal, which, as before, ensures further charging of gold with a negative charge (because the electron current is still larger than the hole current). This leads to a gradual formation of

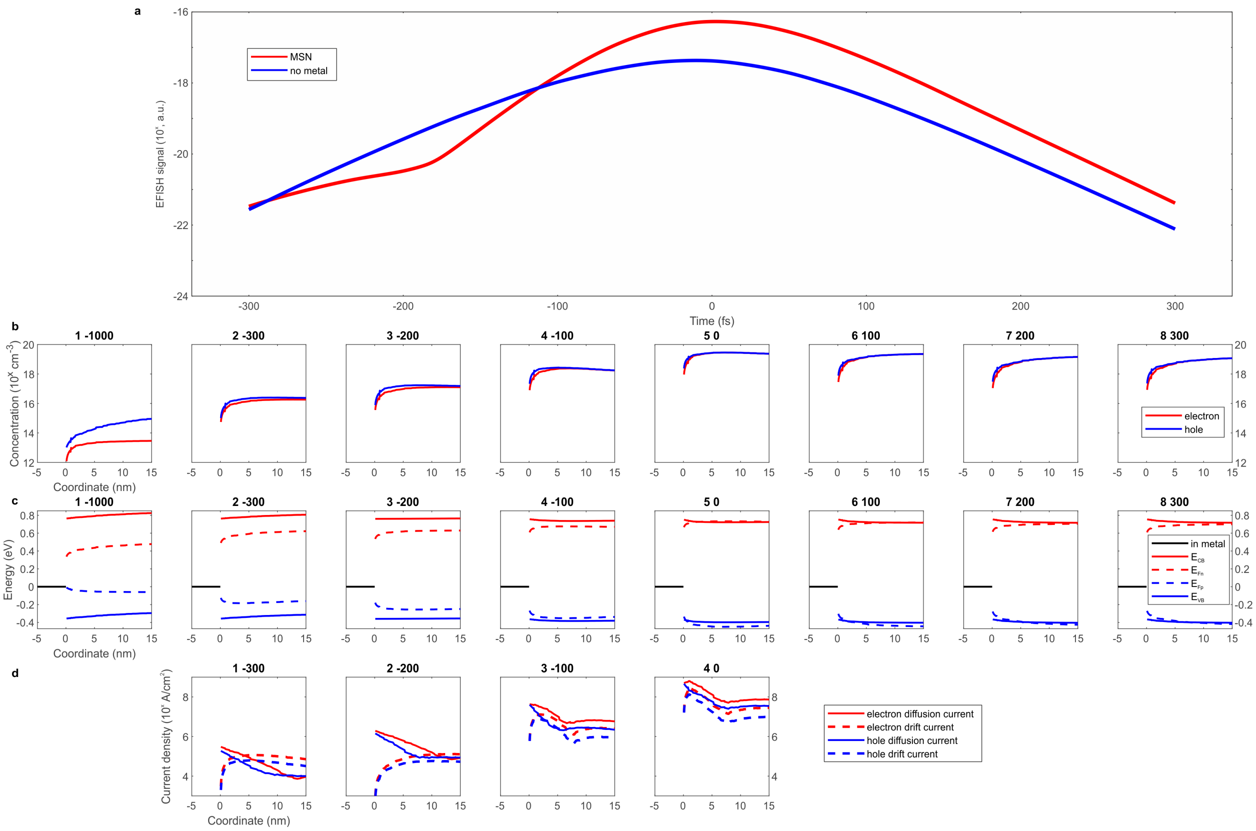

Figure S11: Results of the numerical calculation with the drift-diffusion model for MSN at a metal-semiconductor contact with a time sweep. a, EFISH signal vs. time during interaction with one excitation pulse for a nanostructure with a metal element (i.e., MSN, red line) and without (blue line). b, Electron and hole density distribution near the metal-semiconductor contact. c, Band diagram close to the metal-semiconductor contact area. d, Distribution of drift and diffusion current components for electrons and holes. The negative part on the coordinate axis for b-d corresponds to the area in the metal, and the positive part, to that in the semiconductor. The labels above the figures b-d (in bold) correspond to the time moments in fs. Time 0 fs corresponds to the maximum of the excitation pulse.

a barrier for electrons (-100 fs) and an increase in the drift current. The diffusion current at the interface is also equalized, which stops the flow of uncompensated charges into the metal and an increase in the barrier height. In fact, fom now on, the height of the barrier is almost constant. Additionally, due to the increase of the carrier concentration in silicon, the thickness of the space charge region near the interface decreases, which leads to a decrease in the barrier thickness (from −100 fs to 0 fs). As a consequence, the electric field strength increases, which ensures the growth of the EFISH signal.

The solution with the drift-diffusion model allowed us to plot the distribution of the built-

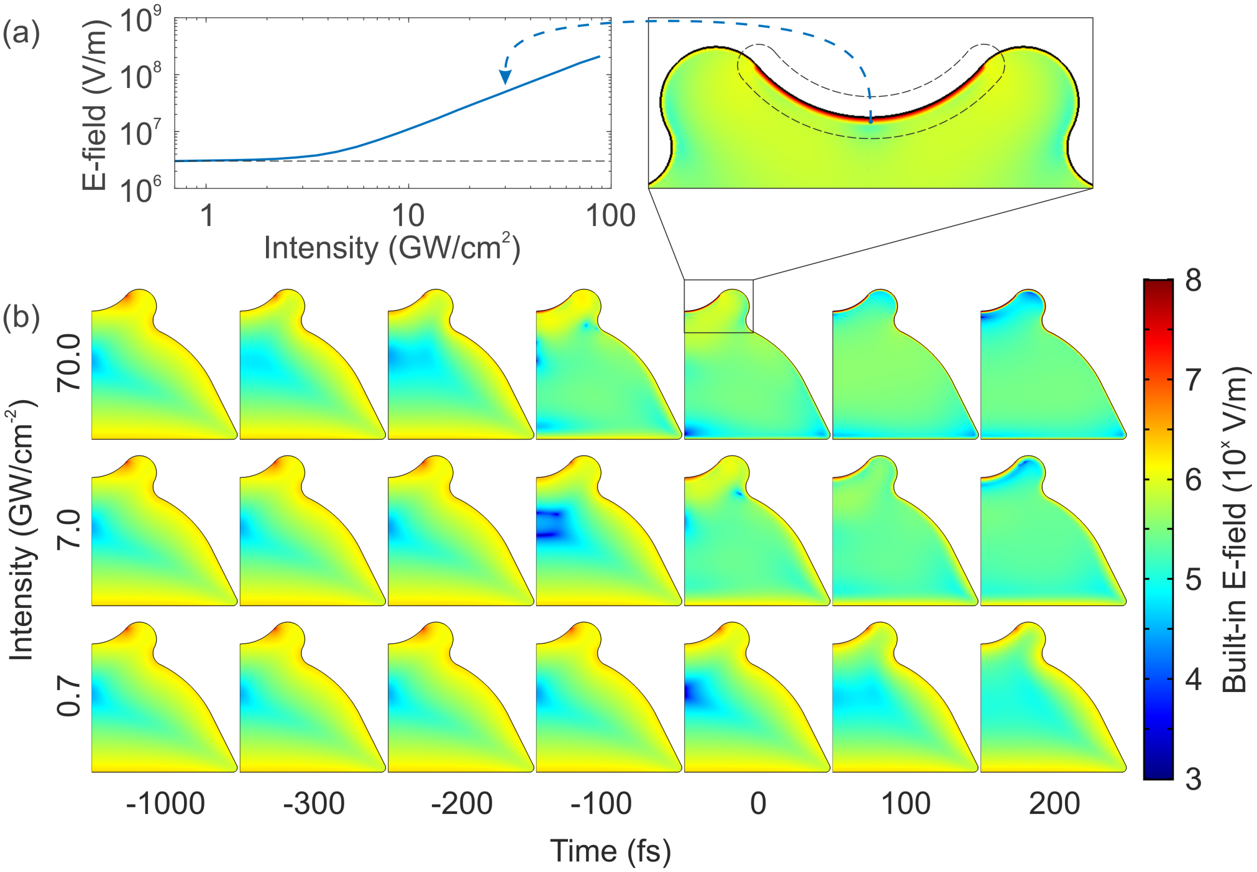

Figure S12: (a) The dependence of the built-in electric field on the excitation power at a point located close to the contact on the side of the semiconductor on the axis of rotation of the structure. (b) Numerical distributions of the built-in field from the drift-diffusion model for a nanostructure in the semiconductor part at different times with respect to the laser pulse ($t = 0$ sec corresponds to the moment of maximum excitation intensity) and different excitation intensities. The color indicates the value of the built-in field on a log-scale.

in field at different excitation powers and at different times during the action of the laser pulse (see Fig. S12b). At threshold power values between two slopes, a field is formed along the MS contact. We monitor the field value at a point in space directly near the contact, and its value vs. on the pump power is shown in Fig. S12a. Under a certain threshold, the field strength does not depend on pumping power up to 10 GW/cm^2. However, as we reach the threshold, the field begins to grow polynomially with increasing excitation and reaches a value on the order of 10^8 V/m. The value is limited from above by the destruction of the nanostructure.

Roughly, for the depletion region in the approximation of one carrier type, one can write

a formula relating its width to the barrier height and doping level as follows:

$$w \approx \sqrt{\frac{2\epsilon\epsilon_0 \Delta\phi}{eN}} \tag{S1}$$

where $\Delta\phi$ is the barrier height, and N is the concentration of carriers (in this case, holes), and the magnitude of the field strength is:

$$E \approx \frac{\Delta\phi}{w} \sim \sqrt{\Delta\phi N} \tag{S2}$$

Since the EFISH signal is proportional to E^2, and the carrier concentration is proportional to the square of the intensity due to the quadratic absorption in silicon for such laser pulse energies, we obtain an estimate for the EFISH signal:

$$I_{EFISH} \sim E^2 I^2 \sim \Delta\phi N I^2 \sim \Delta\phi I^4 \tag{S3}$$

i.e., the functional dependence is higher than the quadratic one. After the time of 0 fs, the intensity of the exciting pulse decreases, and the system gradually relaxes after the end of the pulse (the time is more than +300 fs).

A comparison of this situation with the one with no transport of charge carriers into the metal (Fig.S11a) for the same excitation intensity and generation rate shows that the possibility of charge transfer through the metal-semiconductor interface and its recharging ensures an increase in the EFISH signal and contributes to the non-quadratic part of this dependency.

Optical signal repeatability short of damage-threshold

In the case of our experiments we have estimated the stability of the MSN morphology upon excitation of a SHG signal via changes of a dark-field scattering signal. Indeed, the geometry and material properties of a nanostructure affect how it scatters light. Therefore, changes in

the morphology of the MSN can be traced in the dark-field scattering spectra. This feature was taken into account in the optical setup configuration used in our experiments. On each step of the SHG signal measurements on the excitation intensity variation, the dark-field spectra were measured to mark the absence of the morphology changes. Such opportunities were obtained due to the three channel optical scheme realization (one objective is for signals collection, another one is for the SHG signal excitation, and the last one is for the side illumination (please, see Fig. S13a and Fig. S2)).

The example of such experiments is represented in the Fig. S13. The considered MSN had the dark-field spectrum shown in the Fig. S13d. At the first stage of checking the MSN morphology stability, the time-dependent SHG signal was measured several cycles in a row (Fig. S13b) under the constant value of the excitation intensity (27 GW/cm^2), when a static field is formed at the metal-semiconductor contact. The time interval between measurements

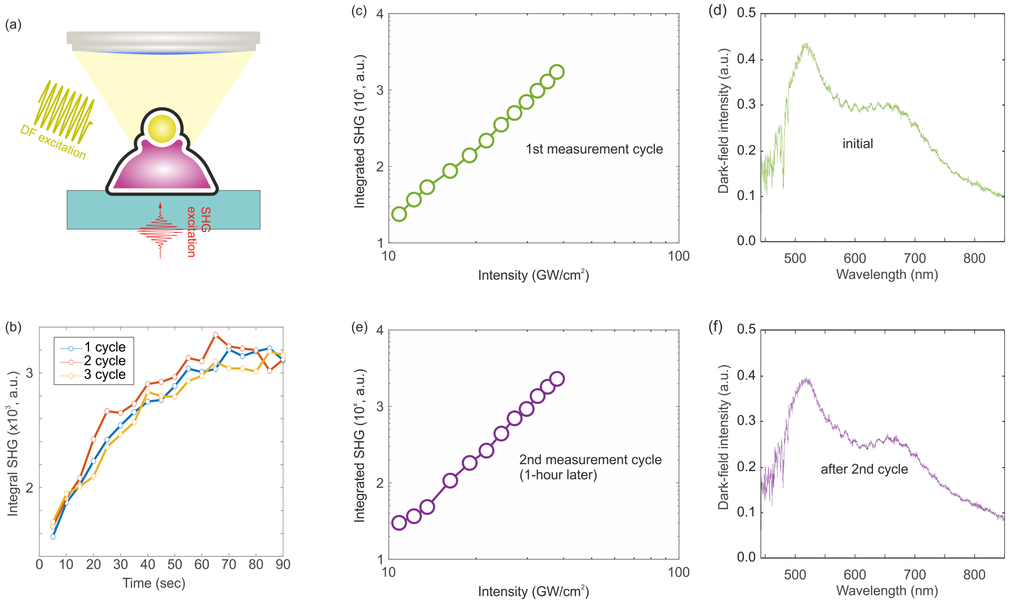

Figure S13: Stability of the MSN during the generation of a nonlinear optical signal. (a) Schematic representation of the setup for SHG with optical verification of morphology stability after several cycles of measurements using dark-field spectroscopy. (b) Time-dependent SHG signal at an excitation intensity of 27 GW/cm^2. Each measurement cycle was performed after a time-delay of 7 min. (c, e) SHG intensity of MSN versus excitation intensity (c) at the 1st and (e) at the 2nd cycle of measurements. The time-delay between cycles is 1 hour. (d, f) Scattering spectra for MSN (d) before and (f) after nonlinear measurements.

was 7 min. This time was sufficient for the system to return to its original state. As can be seen from the figure, the curves repeat each other within the permissible error.

At the second stage, a series of measurements of the SHG signal was performed at different excitation intensities short of 70 GW/cm^2 (Fig. S13c). The dependence in the considered excitation intensity range has a slope close to the 4th order. After a long time interval (almost 1 hour), the series of measurements was repeated (Fig. S13e). The results in the case of the first and second cycles of measurements repeat each other well, which indicates the stability of the nonlinear optical response from the MSN within the allowable excitation intensities. The experiment ends with the last dark-field spectrum (Fig. S13f), which has no significant changes with the initial result (Fig. S13d). Therefore, the MSN morphology is preserved during the generation of the second harmonic.

Details of the numerical calculation of the second harmonic generation signal

We performed the second harmonic simulations in COMSOL Multiphysics by combining two Electromagnetic waves, Frequency domain interfaces corresponding to the fundamental radiation frequency ω and the second harmonic 2ω. In one interface, an incident electromagnetic wave was simulated at the fundamental wavelength, and the second interface corresponded to SHG and was implemented by specifying a nonlinear polarization inside the silicon part of the MSN structure:

$$P_i = \varepsilon_0 \sum_{jkl} \chi^{(3)}_{ijkl} E^{DC}_j E^{\omega}_k E^{\omega}_l \tag{S4}$$

where E^{ω} is the electric field at the pump frequency ω, and E^{DC} is the formed static electric field, which depend on the intensity of the incident radiation $E^{DC} = f(I_{exc})$ (Fig. 4g), $\widehat{\chi}^{(3)}$ is the third order third-order nonlinear susceptibility tensor in Si.[2] Thus, this polarization

serves as a nonlinear source and makes it possible to calculate SHG from the MSN structure integrated into the upper half-space (red line in the Fig. S14) and also into the aperture of the objective used in the experiment (black line in the Fig. S14). Indeed, according to the numerical results, the formation of a static field leads to a fourth degree dependence between the incident intensity and the intensity of the second harmonic for high input values, which is not typical for surface SHG, i.e. the only possible alternative way of second harmonic generation in centrosymmetric Si material. Moreover, calculated static fields reaching about 10^8 V/m within a 1 nm layer (Fig. 4g) and used $\hat{\chi}^{(3)}$ components of about 10^{-19} m^2/V^2

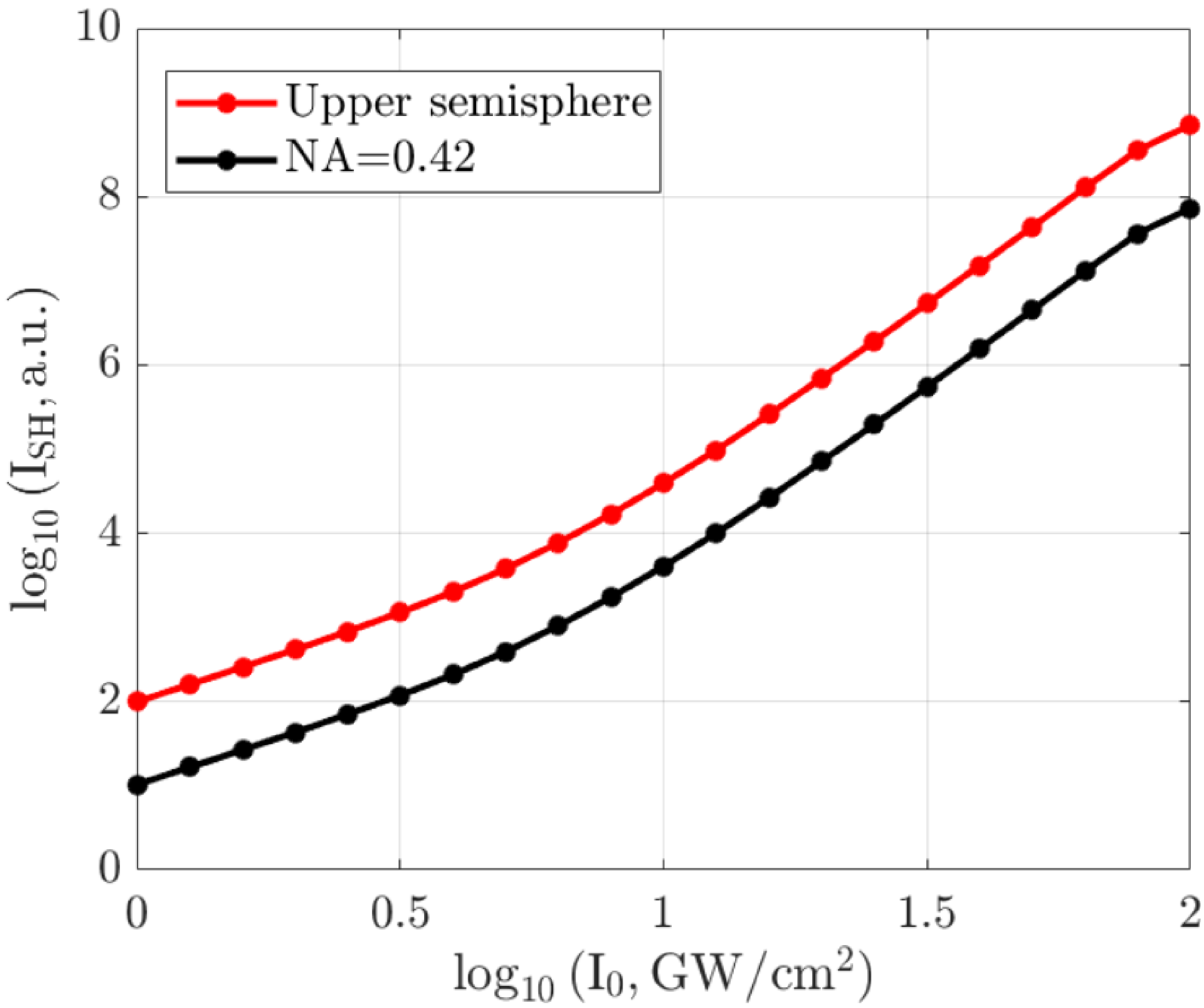

Figure S14: Calculated dependence of the SHG signal intensity integrated over the upper hemisphere (red line) and numerical aperture 0.42 (black line) on the incident laser intensity I_0 for the MSN structure on a log-log scale.

contribute to $\chi^{(2)} = \chi^{(3)} E^{DC} = 10^{-19}\ m^2/V^2 \cdot 10^8\ V/m \cdot 10^{-9}\ m = 10^{-20}\ m^2/V$, which is an order of magnitude higher than the surface second-order nonlinear susceptibility $\chi^{(2)}_{surf} = 10^{-21}\ m^2/V$ in this material.[3] Thus, we once again emphasize the decisive role of the EFISH in the experimentally observed signal and its non-quadratic behavior for high laser intensities.

References

(1) Makarov, S. V.; Petrov, M. I.; Zywietz, U.; Milichko, V.; Zuev, D.; Lopanitsyna, N.; Kuksin, A.; Mukhin, I.; Zograf, G.; Ubyivovk, E.; Smirnova, D. A.; Starikov, S.; Chichkov, B. N.; Kivshar, Y. S. Efficient second-harmonic generation in nanocrystalline silicon nanoparticles. *Nano Letters* **2017**, *17*, 3047–3053.

(2) Wynne, J. Optical third-order mixing in GaAs, Ge, Si, and InAs. *Physical Review* **1969**, *178*, 1295.

(3) Gavrilenko, V.; Rebentrost, F. Nonlinear optical susceptibility of the surfaces of silicon and diamond. *Surface science* **1995**, *331*, 1355–1360.
